# *In vivo* outer hair cell gene editing ameliorates progressive hearing loss in dominant-negative *Kcnq4* murine model

**DOI:** 10.7150/thno.67781

**Published:** 2022-02-28

**Authors:** Byunghwa Noh, John Hoon Rim, Ramu Gopalappa, Haiyue Lin, Kyu Min Kim, Min Jin Kang, Heon Yung Gee, Jae Young Choi, Hyongbum Henry Kim, Jinsei Jung

**Affiliations:** 1 Department of Otorhinolaryngology, Graduate School of Medical Science, Brain Korea 21 Project, Yonsei University College of Medicine, Seoul 03722, Republic of Korea; 2 Institute for Yonsei Ear Science, Seoul 03722, Republic of Korea; 3 Department of Laboratory Medicine, Yonsei University College of Medicine, Seoul 03722, Republic of Korea; 4 Department of Pharmacology, Graduate School of Medical Science, Brain Korea 21 Project, Yonsei University College of Medicine, Seoul 03722, Republic of Korea; 5 Severance Biomedical Science Institute, Yonsei University College of Medicine, Seoul, Republic of Korea; 6 Center for Nanomedicine, Institute for Basic Science, Seoul 03722, Republic of Korea; 7 Yonsei-IBS Institute, Yonsei University, Seoul 03722, Republic of Korea

**Keywords:** DFNA2, outer hair cell, KCNQ4, Cas9, gene editing

## Abstract

Outer hair cell (OHC) degeneration is a major cause of progressive hearing loss and presbycusis. Despite the high prevalence of these disorders, targeted therapy is currently not available.

**Methods:** We generated a mouse model harboring *Kcnq4*^W276S/+^ to recapitulate DFNA2, a common genetic form of progressive hearing loss accompanied by OHC degeneration. After comprehensive optimization of guide RNAs, Cas9s, vehicles, and delivery routes, we applied *in vivo* gene editing strategy to disrupt the dominant-negative allele in *Kcnq4* and prevent progressive hearing loss.

**Results:** *In vivo* gene editing using a dual adeno-associated virus package targeting OHCs significantly improved auditory thresholds in auditory brainstem response and distortion-product otoacoustic emission. In addition, we developed a new live-cell imaging technique using thallium ions to investigate the membrane potential of OHCs and successfully demonstrated that mutant allele disruption resulted in more hyperpolarized OHCs, indicating elevated KCNQ4 channel activity.

**Conclusion:** These findings can facilitate the development of targeted therapies for DFNA2 and support the use of CRISPR-based gene therapy to rectify defects in OHCs.

## Introduction

Hearing loss is a common sensory disorder 1 that affects 466 million people (432 million adults and 34 million children), or approximately 5% of the world's population 2. In particular, the risk of hearing impairment increases with age, and the number of individuals with age-related hearing loss (ARHL) is growing each decade 3, while there is currently no treatment for ARHL. The main causes of ARHL are aging, loud noise exposure, genetic variants, and exposure to ototoxic drugs or chemicals that cause damage to the cochlea 4,5. While numerous genetic and environmental risk factors for ARHL development have been reported 6, acoustic noise exposure over a person's lifetime is considered the primary cause of ARHL 7,8. The extent to which ARHL is attributable to genetic factors is still unclear 6; however, it has been reported that alterations in genes that encode potassium channels located in the cochlea may influence individual susceptibility to noise exposure and increase the risk of developing ARHL 9,10.

The pathomechanism of ARHL involves metabolic and sensory damage along with neurodegeneration in the outer hair cells (OHCs), ribbon synapses, basement membrane, and stria vascularis 11-14. One of the mechanistic causes of ARHL is OHC degeneration, which can occur with aging and results in the loss of otoacoustic emission responses 15,16. In humans, the occurrence and extent of age-related OHC loss and ARHL are affected by genetic variants of KCNQ4, which encodes a voltage-gated potassium channel (Kv7.4) that is highly expressed in the basolateral membrane of OHCs and mediates the M-potassium current 17,18. Methods and technologies that restore KCNQ4 expression and function in OHCs may be valuable for the prevention and treatment of ARHL that results from *KCNQ4* variants.

A high potassium concentration in the cochlear endolymph is fundamental for endocochlear potential, which in turn is crucial for the maintenance of hearing function 19,20. Potassium influx into the OHCs leads to electromechanical sound amplification, and rapid potassium efflux via Kv7.4 on the basolateral surface results in repolarization of the OHCs and recycling of potassium to the endolymph 19. *Kcnq4*^-/-^ mice exhibit progressive hearing loss accompanied with selective OHC degeneration 18. Further, Kv7.4 is associated with autosomal dominant non-syndromic hearing loss, called deafness non-syndromic autosomal dominant 2 (DFNA2) 21. Kv7.4 is composed of pore-forming tetramer subunits, and defects in even a single subunit can impair the channel function of the tetramer, which underlies the development of hearing loss in patients with a heterozygous variant in *KCNQ4*. While most variants have a dominant-negative effect on the wild-type (WT) Kv7.4 22, several variants located in the N-terminal domain are associated with haploinsufficiency 21,23.

Genome editing technologies, including Cas9 nucleases, base editors, and prime editors, are emerging as tools for the treatment of various diseases caused by genetic defects 24. In particular, Cas9 nucleases are effective in disrupting dominant-negative alleles owing to their high capability of selective inactivation, whereas base and prime editors are useful for rescuing recessive loss-of-function variants 25. However, *in vivo* therapeutic gene editing for deafness is facing numerous challenges, including the selection of suitable vehicles and delivery routes for efficient and safe delivery of the gene editing system 26,27. Recently, *in vivo* gene editing using Cas9 nuclease or base editor was successfully applied to restore hearing in mice with transmembrane channel-like 1 gene (*Tmc1*) variants 28-30. However, the studies also showed the limits of the Cas9 nuclease and base editor in hearing restoration regarding long-lasting and meaningful efficacy. Furthermore, given the extremely heterogeneous pathogenesis of hearing loss, whether *in vivo* gene editing is a feasible strategy and applicable to other disease models of deafness requires validation. The options for animal models of deafness for gene editing are currently limited, which hampers the development of *in vivo* applications. Specifically, there has been no attempt to restore hearing function in animal models in which deafness is attributed solely to damaged OHCs.

In this study, we employed *in vivo* gene editing using a Cas9 nuclease system with the aim to ameliorate progressive hearing loss resulting from OHC degeneration using a newly established murine model with a dominant-negative effect of a missense variant (p.W276S) in *Kcnq4*. We set up the strategy to disrupt the dominant-negative allele in *Kcnq4* using SpCas9 nuclease. In addition, the efficacy, safety, and delivery route of the Cas9 nuclease system were optimized to improve the feasibility of *in vivo* genome editing for the treatment of DFNA2.

## Results

### Generation of the *Kcnq4* mutant mouse model and the baseline characterization for auditory function

**Figure 1:**
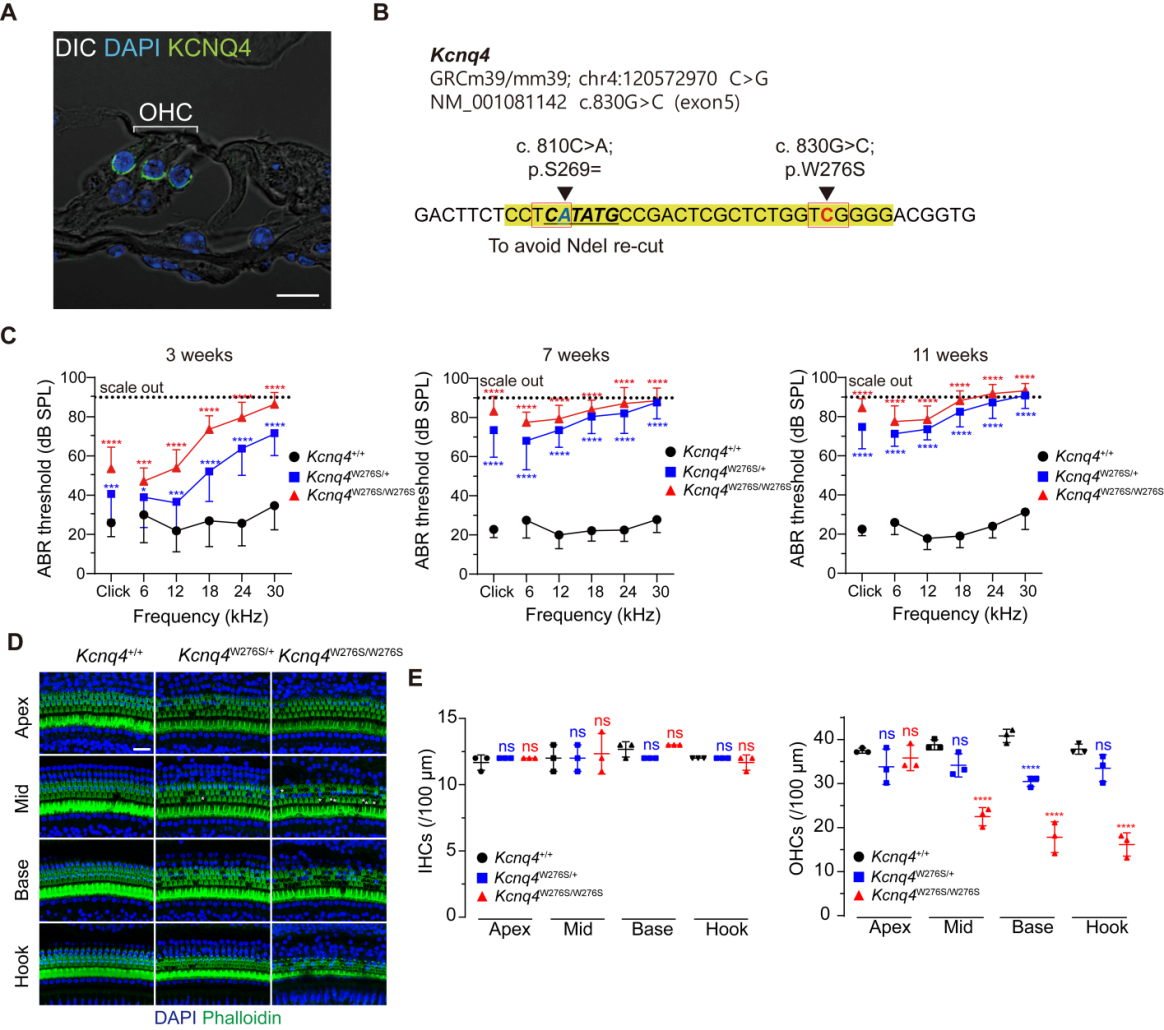
** Characterization of the *Kcnq4* knock-in (c.830G>C) mouse model.** (**A**) Immunostaining with anti-Kcnq4 antibody was performed in a 3-week-old wild-type (WT) mouse. Kcnq4 was exclusively expressed in the basolateral surface of outer hair cells (OHCs) in the organ of Corti of the inner ear. Scale bar, 10 µm. (**B**) Sequence data for the *Kcnq4* mutant mouse model used. A pathogenic missense variant in *Kcnq4* (c.830G>C) was targeted for gene editing. Prior to target variant, an additional synonymous variant (c.810C>A) was introduced for the identification of the original mutant allele after Cas9 editing at the target variant site and to prevent the NdeI restriction enzyme from cutting within this position, enabling the use of NdeI for genotyping of the mutant allele. (**C**) Characteristics associated with auditory function in *Kcnq4* knock-in mice. *Kcnq4*^+/+^ (black, *n* = 8 mice), *Kcnq4*^W276S/+^ (blue, *n* = 12 mice), and *Kcnq4*^W276S/W276S^ (red, *n* = 7 mice). *p < 0.05; ***p < 0.001; ****p < 0.0001 by two-way ANOVA followed by Bonferroni's correction for multiple comparisons. (**D**) Whole-mount images of the cochlea at the apex, mid, base, and hook regions from 3-week-old WT, *Kcnq4*^W276S/+^, and *Kcnq4*^W276S/W276S^ mice. Immunostaining was performed with DAPI (blue) and phalloidin (green). Scale bar, 20 μm. **(E)** Numbers of live inner hair cells (IHCs) and OHCs in images indicated in (D) (n = 3 mice in each group). ****p < 0.0001; ns, not significant by two-way ANOVA followed by Bonferroni's correction for multiple comparisons.

We screened the mouse models to identify those with the pathology of deafness in the OHCs for application of gene editing and found that DFNA2 caused by the mutations in *KCNQ4* is the best candidate for several reasons, which include the following: 1) variants in *KCNQ4* are associated with age-related and noise-induced hearing loss 9,31; 2) DFNA2 is characterized by progressive hearing loss, potentially providing a broad range of ages over which successful therapy may be possible; 3) Kv7.4 is primarily expressed both at the basolateral surfaces of OHCs (**Figure 1A**) and in the spiral ganglion and is transiently observed in IHCs (**Figure S1B**). Therefore, *KCNQ4* can be used to evaluate the efficacies of gene editing system delivery and gene editing in progressive hearing loss such as DFNA2 caused by degeneration of OHCs 17.

Among the more than 40 DFNA2-associated *KCNQ4* variants, we selected *KCNQ4* c.827G>C (NM_004700.3, p.W276S) as a candidate for *in vivo* gene editing because this variant is common worldwide 21,32,33. Thus, knock-in mice harboring the corresponding *Kcnq4* c.830G>C (NM_001081142) allele and a silent variant at nucleotide position 810 (c.810C>A; p.S269=) were generated (**Figure 1B**). The silent variant not only facilitated genotyping by rendering only *Kcnq4* c.830G>C susceptible to NdeI but also allowed discrimination of edited mutant alleles containing insertion and deletion (indel) variants from WT alleles affected by Cas9 single-guide RNA (sgRNA) in *in vivo* gene editing experiments.

We found that *Kcnq4*^W276S/W276S^ (homozygote) and *Kcnq4*^W276S/+^ (heterozygote) mice distinctly exhibited moderate hearing loss as indicated by an increased auditory brainstem response (ABR) threshold at 3 weeks after birth (**Figure 1C**). Progressive hearing loss phenotype was validated in follow-up hearing function tests performed at 7 and 11 weeks after birth. Hearing loss was attributed to the degeneration of OHCs, particularly in the high-frequency region of the cochlea (**Figures 1D-E**). The morphology of stereocilia in surviving hair cells of the *Kcnq4*^W276S/+^ mice was normal at 4 weeks of age (similar to that in WT mice; **Figure S1**). Hearing thresholds at all frequencies reached nearly scale-out levels at 7 weeks of age (**Figure 1C**).

### Optimization of the targeting efficacy of the Cas9-sgRNA system for Kcnq4 c.830G>C

**Figure 2:**
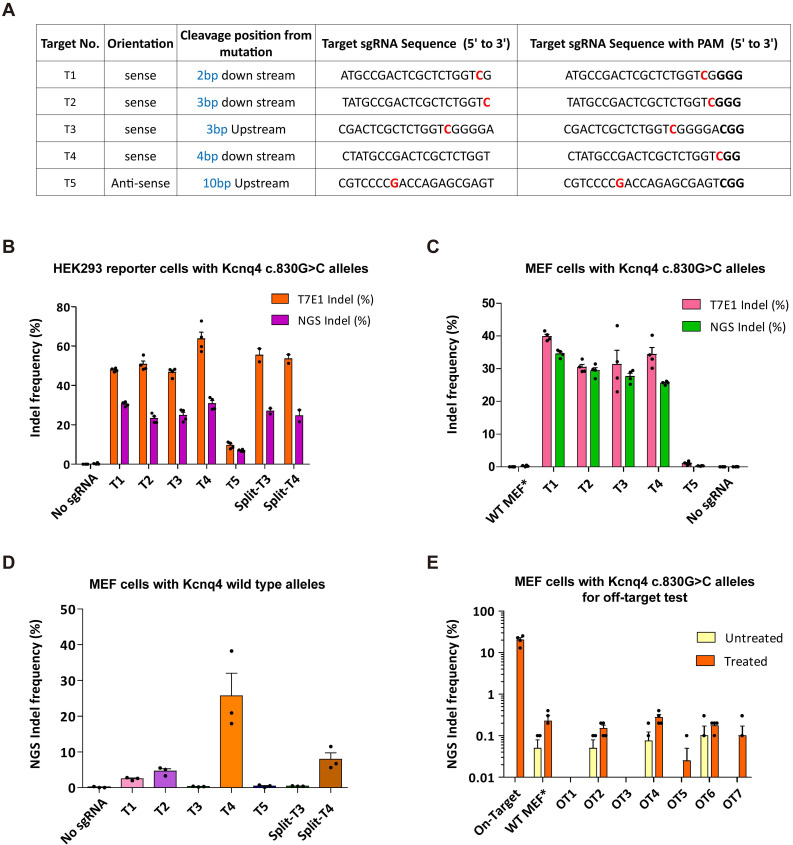
** Optimization of sgRNA for *in vivo* gene editing.** (**A**) Profiles of five sgRNA candidates. Protospacer-adjacent motif (PAM) sequences are shown in bold. Red colors refer to the targeting missense variant. (**B-C**) On-target efficacy of sgRNA candidates determined in HEK293 reporter cells and MEFs harboring the *Kcnq4* target mutant allele (c.830G>C) by T7E1 assay and deep sequencing analysis (n = 4 in each group). (**D**) Off-target activity for sgRNA candidates was evaluated in MEFs with *Kcnq4* WT alleles using deep sequencing (n = 3 in each group). **(E)** Indel frequencies of the selected sgRNA-T3 was evaluated in the seven top-ranked genome wide off-target sites. In MEFs harboring the *Kcnq4* target mutant allele (c.830G>C), off-target activities of T3 sgRNA and SpCas9 (treated group, n = 3 in each column) on the seven top-ranked off-target sites were compared to those without sgRNA and Cas9 (untreated group, n = 2 in each column). MEF, mouse embryo fibroblast; NGS, next-generation sequencing; Indel, insertion and deletion; WT, wild-type; WT-MEF*, MEFs with *Kcnq4* WT alleles

We chose the strategy of disrupting the DFNA2-related mutant allele c.830G>C, which has a dominant-negative effect on the WT allele in *Kcnq4*^W276S/+^ mice 22. All possible sgRNA target sequences with a protospacer-adjacent motif (PAM; 5′-NGG-3′) downstream of the variant locus were designed (**Table S1**) in order to select active and efficient sgRNAs to target the *Kcnq4* variant (c.830G>C). Gene editing scores for all sgRNAs were determined using the deepSpCas9 prediction program 34, which indicated that the combination of WT SpCas9 (*Streptococcus pyogenes* Cas9) and sgRNA-T3 was high efficient among the various types of SpCas9s (**Table S1**). We selected five sgRNAs with high efficacy in all Cas9 variant types for evaluating *in vitro* efficacy (**Figures 2A** and** S2A).** To elucidate the activity of the sgRNAs, we generated a reporter cell line expressing the *Kcnq4* variant (c.830G>C) target sequence with a dual-fluorescent reporter construct 35,36. Both the SpCas9 and sgRNAs were transfected into human embryonic kidney (HEK)293 reporter cells with *Kcnq4* c.830G>C mutant alleles (**Figure S2B-C**). The presence of the reporter was assessed using the red fluorescent protein (RFP) signal, while the GFP was only identified as present if the Cas9 nuclease and sgRNA generated indels, which rendered the *GFP* in frame (**Figure S2D**). All sgRNAs tested induced GFP fluorescence, indicating that they were effective (**Figure S2E**).

Next, HEK293 reporter cells with *Kcnq4* c.830G>C mutant alleles were subjected to a T7 endonuclease I (T7E1) assay, in which the cleavage activity of the endonuclease was revealed by the presence of cleaved bands, and deep sequencing analysis. Indel frequencies at the target site for the selected sgRNAs (T1-T5) ranged from 9.6%-63.7% and 7%-30.8% according to the T7E1 assay and deep sequencing analyses, respectively (**Figures 2B** and** S3A**); T5 sgRNA showed the lowest indel frequency (9.6% via T7E1 and 7.0% via deep sequencing). For the confirmatory test, we performed gene editing efficacy and indel generation for selected sgRNA using mouse embryonic fibroblasts (MEFs) with c.830G>C mutant alleles generated from *Kcnq4* c.830G>C homozygote mouse **(Table S2)**. The results showed similar editing efficacies to those of HEK293 reporter cells (**Figures 2C** and** S3B**).

To determine the WT allele cleavage (off-target) activity of the selected sgRNAs, we transiently transfected Neuro2a cells harboring *Kcnq4* WT allele with SpCas9 and sgRNAs (T1-T5). T7E1 assays revealed that T1 (9%), T3 (0%), and T5 (0%) sgRNAs had negligible off-target activity at the WT target allele, while T2 (41%) and T4 (47%) exhibited off-target activity **(Figure S3C)**. In MEFs with *Kcnq4* WT alleles from *Kcnq4* WT homozygote mouse, the off-target effect was remarkably low in T3 and T5 sgRNAs **(Figure 2D)**. Furthermore, we screened potential genome-wide off-target sites using Cas-OFFinder algorithm 37, selected top-ranked seven loci, and evaluated the off-target effect of T3 sgRNA in the MEFs with *Kcnq4* c.830G>C alleles **(Table S3)**. The results showed that the off-target effects on these loci were negligible (<0.4%) compared to the on-target effect of T3 sgRNA (**Figure 2E**). Finally, sgRNA-T3 was selected for subsequent *in vivo* gene editing as it showed no cleavage activity on the WT target locus, while exhibiting high cleavage activity for the c.830G>C variant target sequence.

To examine the efficacy of the dual-adeno-associated virus (AAV) system carrying SpCas9 and sgRNA, we first designed split SpCas9 (due to large coding size of SpCas9) plasmids and gRNAs (**Figure S4A-B**). For transgene packaging, we used the capsids AAV2/Anc80L65 for delivery into the cochlear hair cells because of their high transduction efficacy in inner hair cells (IHCs) and OHCs 38. We compared the *in vitro* efficacy of split SpCas9 (N- and C-terminal) with full version of SpCas9. Each type of SpCas9 was transfected into the HEK293 reporter cells with *Kcnq4* c.830G>C alleles. The on-target cleavage activity of split SpCas9 was comparable to that of non-split SpCas9 (**Figures 2B** and** S3D**). The off-target cleavage activity of split SpCas9 in MEF and Neuro2a cells harboring *Kcnq4* WT allele was also comparable to that of full version of SpCas9, regardless of the type of sgRNA used (**Figures 2D** and** S3E**). Next, we produced dual AAVs and evaluated the AAV titer, calculated as genome copies per milliliter. The genome copies were determined by quantitative (q)PCR using primers specific for the inverted terminal repeats of the virus. The final titer of the combined AAVs injected into one cochlea was estimated to be 1.00 × 10^9^ genome copies/µL (**Figure S4C**).

To determine the best delivery route, we investigated the *in vivo* efficacy of viral injection via the round window (RW), scala media (SM), posterior semi-circular canal (PSCC), and utricle, using AAV2/Anc80L65-eGFP (**Figure S5A**). As previously reported 38, Anc80L65 capsid showed a high efficient transduction rate in the OHCs via all delivery routes, while the utricle route resulted in the highest eGFP expression levels at all frequency regions (**Figure S5B**). When we added SpCas9 packaged to the Anc80L65 capsid via SM at P2, SpCas9 was detected in the OHCs and IHCs of the AAV-injected ear, whereas Cas9 in the contralateral ear and the ear of non-injected mouse was marginal and absent, respectively **(Figure S6)**. Hearing loss was generally not induced in WT mice when AAV complex and a ribonucleotide complex (RNP) injection via cochleostomy was performed appropriately (**Figure S7A-B**).

### Hearing restoration of Kcnq4 p.W276S mice via CRISPR/Cas9 gene editing

**Figure 3:**
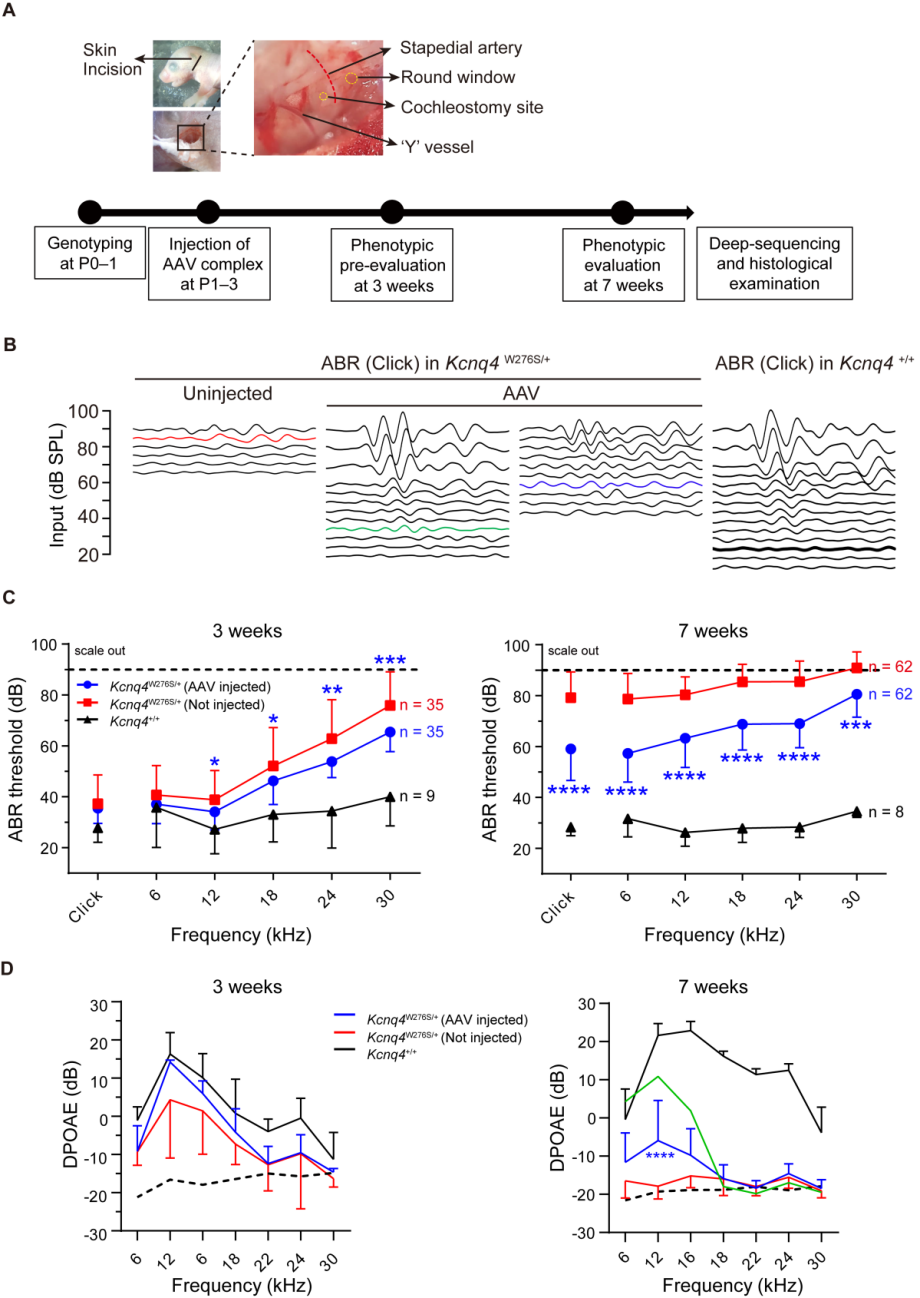
** Hearing restoration by AAV injections in *Kcnq4^W276S/+^* mice.** (**A**) Experimental workflow of injection procedures to examine the hearing phenotype and determine the gene editing efficacy. P, postnatal day. (**B**) Auditory brainstem response (ABR) waveform patterns of the AAV-injected cochlea and contralateral non-injected cochlea from 7-week-old *Kcnq4* mutant and WT mice (control). The highest (green) and median (blue) recovery degrees are shown. (**C**) Comparative analyses of ABR thresholds across all frequencies in AAV-injected and non-injected cochlea of mutant and WT control mice at 3 and 7 weeks. Statistical comparisons were performed between AAV-injected and non-injected mice. *p < 0.05, **p < 0.01, ***p < 0.001, and ****p < 0.0001 by two-way ANOVA followed by Bonferroni's correction for multiple comparisons. (**D**) Distortion-product otoacoustic emission (DPOAE) thresholds in AAV-injected mutant (n = 18), non-injected (n = 18) mutant, and WT (n = 6) mice at 3 and 7 weeks. The highest value in AAV-injected mice at 7 weeks depicts in green color. Dashed black line refers to the level of noise floor. ****p < 0.0001 by two-way ANOVA followed by Bonferroni's correction for multiple comparisons.

After injecting the appropriate AAV complexes into *Kcnq4*^W276S/+^ mice before or at P3, hearing restoration was evaluated based on ABRs and distortion-product otoacoustic emissions (DPOAEs) at 3 and 7 weeks after injection (**Figure 3A**). **Figure 3B** shows representative ABR traces in response to varying sound intensities of 6-kHz tone bursts at 7 weeks after injection. In AAV-injected ears of *Kcnq4*^W276S/+^ mice, ABR thresholds at 12-, 18-, 14-, and 30-kHz were significantly lower than those in non-injected ears at 3 weeks (**Figures 3C, left**). At 7 weeks, the ABR thresholds at all frequencies were significantly better in AAV-injected ears than non-injected ears (**Figures 3C, right**). In addition, P1 amplitude and P1 latency in AAV-injected ears were significantly improved compared with those in non-injected ears (**Figure S8A-C**). In addition, we confirmed that only SpCas9 injection without sgRNA had no effect on the hearing threshold, indicating that the hearing restoration was dependent on the targeted sgRNA of *Kcnq4* c.830G>C allele (**Figure S9**). The DPOAE amplitude in AAV-injected ears was significantly increased compared to that in non-injected ears only at frequencies corresponding to the 12-kHz region (**Figure 3D**). However, the difference of hearing thresholds between AAV-injected and non-injected ears decreased at 11 weeks (**Figure S10**).

The outcomes of hearing restoration with AAV and RNP injection via *in vivo* intracochlear delivery were compared by generating an RNP complex to explore their *in vitro* and *in vivo* applications. Briefly, we delivered Cas9-sgRNA RNP complexes at different Cas9:sgRNA ratios into the HEK293 reporter cells with *Kcnq4* c.830G>C mutant alleles, using cationic lipid Lipofectamine 2000 for delivery, as it showed the best efficacy in a previous study (**Figure S11A-B**) 28. The cleavage activity at the target loci, as analyzed by deep sequencing, ranged from 10% to 32% according to the different Cas9-sgRNA complex combinations delivered into the cells (**Figure S11B**). An optimized RNP mixture (1.5 μg of SpCas9 and 1.0 μg of sgRNA) with the highest indel efficacy (32.2%) was used for subsequent *in vivo* studies. In the RNP-injected mice, the improvement of ABR thresholds was comparable to that in the AAV-injected mice (**Figure S11C**). DPOAE amplitudes in RNP vehicle-injected ears were significantly improved in the 12-kHz region (**Figure S11D**). Taken together, the administration of both AAV and the RNP vehicle partially restored hearing function in *Kcnq4*^W276S/+^ mice.

### Gene editing efficacy in mice with hearing restoration

**Figure 4:**
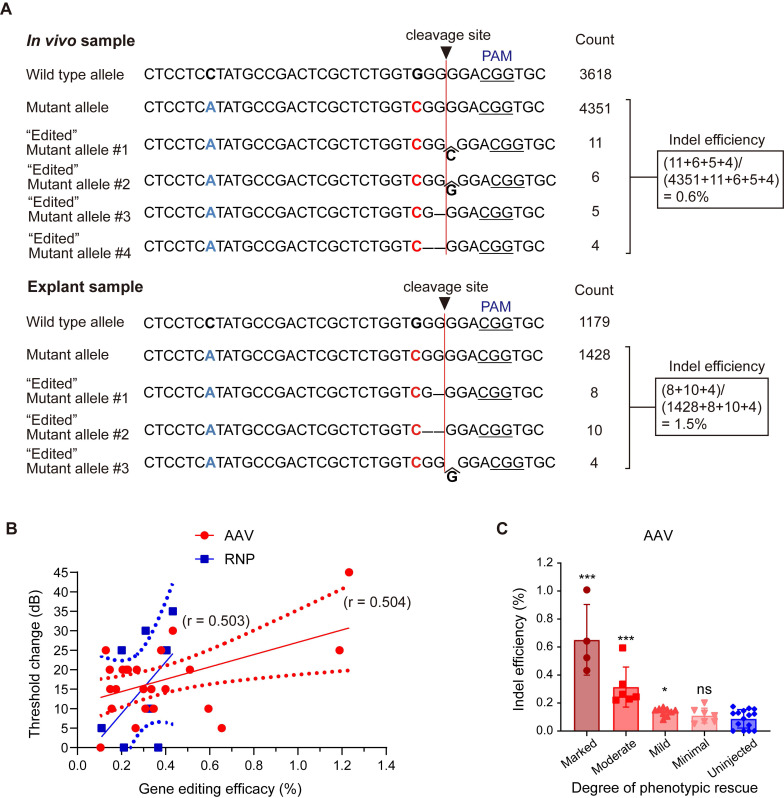
** Evaluation of *in vivo* gene editing efficacy and hair cell survival after AAV injection in *Kcnq4^+/W276S^ mice.*** (**A**) Allele-specific gene editing efficacy obtained through deep sequencing in *in vivo* samples following AAV injection and explant samples following AAV treatment. (**B**) Correlation between gene editing efficacy and the degree of hearing improvement based on ABR threshold shift levels at click sound. Gene editing efficacy showed a statistically significant correlation trend (r = 0.504, *p* < 0.01 by correlation analysis) with ABR threshold shift by AAV injection, but not RNP injection (r = 0.503, p > 0.05 by correlation analysis) (**C**) Gene editing efficacy according to the degree of hearing improvement based on ABR threshold shift levels. The degree of hearing improvement was classified into one of the four subgroups based on the average dB differences between injected and contralateral non-injected cochlea at five frequencies (6, 12, 18, 24, and 30 kHz), and improvement was categorized as follows: marked, ≥15 dB; moderate, 10-14 dB; mild, 5-9 dB; and minimal, ≤5 dB). *p < 0.05; ***p < 0.001; ns, not significant by one-way ANOVA followed by Bonferroni's multiple comparisons.

To estimate *in vivo* gene editing efficacy from the deep sequencing data, allele-specific indel analysis was performed in mice with hearing restoration. When the sample of the phenotypically best-rescued mouse was compared with an explant sample, 0.6% mutant alleles were disrupted in AAV-injected mice, generating diverse indels (similar to explant samples treated with AAV, 1.5%; **Figure 4A**). Reproducible and relevant sequenced fragments having out-of-frame indels at the target cleavage site confirmed the validity of *in vivo* genome editing. Furthermore, the gene editing efficacy in the injected ears correlated with ABR threshold changes after AAV injection (r = 0.504, *p* < 0.01) and RNP injection (r = 0.503, *p* > 0.05) (**Figure 4B**). Next, the indel efficacy (%) was matched with the degree of hearing improvement based on the average differences in ABR thresholds between the injected and contralateral non-injected cochlea (hearing improvement was categorized as follows: marked group: ≥ 15 dB; moderate group: 10-14 dB; mild group: 5-9 dB; and minimal group: ≤ 5 dB). A significant correlation was observed in the marked, moderate, and mild groups among the AAV-injected mice (**Figure 4C**), while no correlation was detected among the RNP-injected mice (data not shown).

### Effect of CRISPR/Cas9-induced disruption of mutant alleles on cochlear function

Next, we examined whether hearing improvement by gene editing results from increased viability of OHCs. We found that OHC viability in *Kcnq4*^W276S/+^ mice with improved hearing thresholds after AAV injection was not significantly different from that in non-injected mice at age of 7 weeks (**Figures S12A** and** 12B**). Similarly, there were no differences in Kv7.4 expression in OHCs (**Figure S12C**), the extent of neurofilament innervation into hair cells (**Figure S12D** and** S12E**), and the number of spiral ganglion neurons (**Figures S13A** and** S13B**) between the AAV-injected and non-injected groups at 7 weeks after injection. Additionally, no morphological differences were observed in the stereocilia on IHCs and OHCs between the two groups (**Figure S13C**). Therefore, hearing restoration by the CRISPR/Cas9 system could not be explained by improved survival of hair cells and neurons.

**Figure 5:**
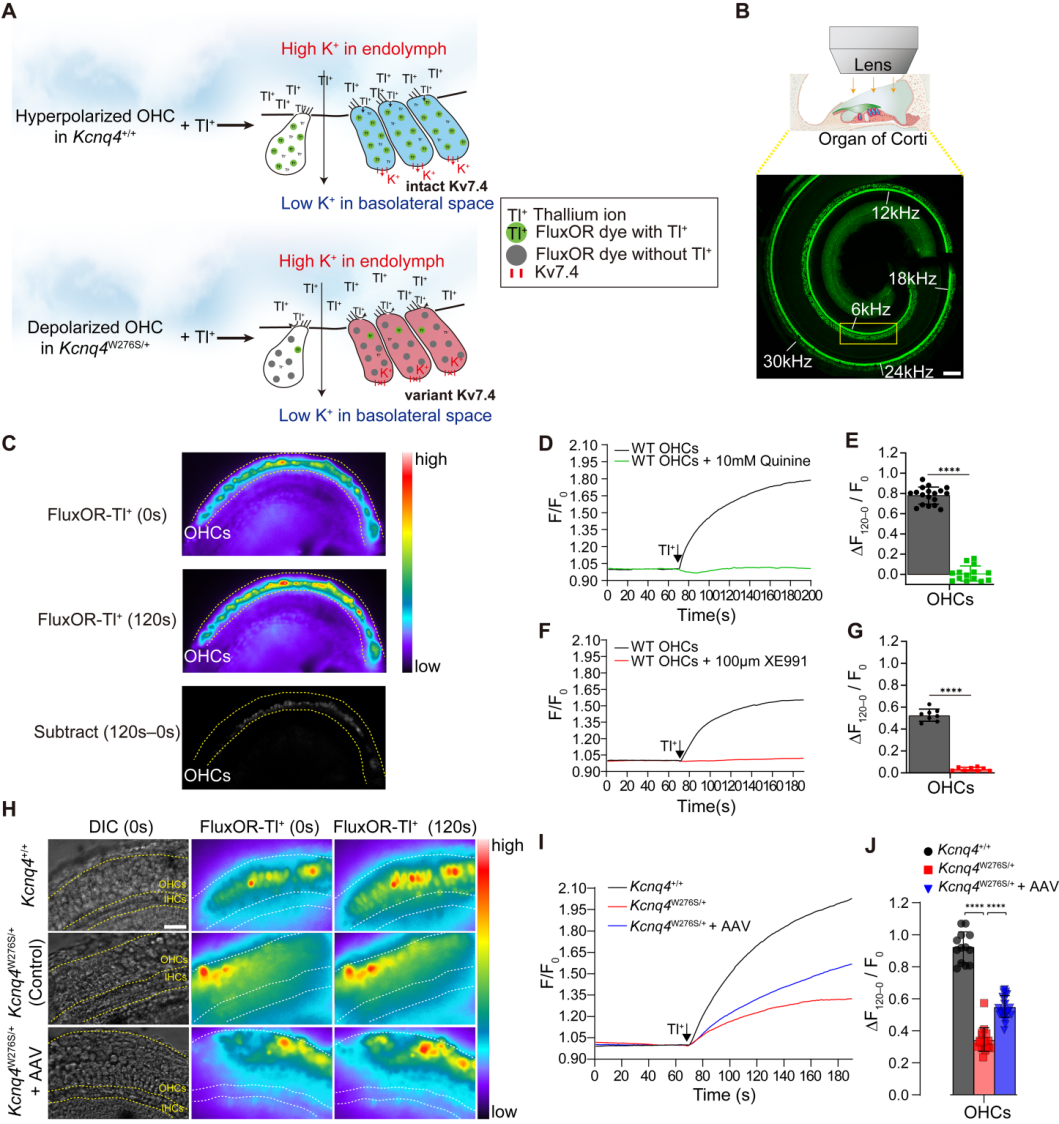
** Mechanisms responsible for hearing restoration in *Kcnq4^W276S/+^* mice upon gene editing by AAV injection.** (**A**) Graphical illustration of the thallium (Tl^+^) flux assay performed in the organ of Corti of 7-week-old WT (top) and *Kcnq4* mutant (bottom) mice. (**B**) Uncapped cochleae were incubated with the dye FluxOR (excitation/emission, 490/525 nm), which is sensitive to thallium ion concentration, and visualized using confocal microscopy. Tonotopic regions are marked with the corresponding frequencies (white). Green color in the cochlear turn refers to phalloidin staining. A Tl^+^ flux assay was performed in the 6-kHz region under a microscope. Scale bar, 100 μm. (**C**) FluxOR fluorescence images of the organ of Corti (apex) of a 7-week-old WT mouse obtained before the apical addition of Tl^+^ (0 s) and after 120 s. Note that the regions of the outer hair cells (OHCs) have prominent color changes. Subtract image refers to the image calculated by [Image 120 s - Image 0 s]. Pseudo colors indicate the intensity of the fluorescent signal. (**D, E**) Fluorescence intensity values (F) relative to the baseline intensity (F_0_, at the time point of Tl^+^ addition) were evaluated in the 6 kHz region of the cochlea of 7-week-old WT mice (D). ΔF_0-120_/F_0_ refers to the change in fluorescent intensity from 0 to 120 s normalized to F_0_ (E). Quinine (10 mM), which inhibits the mechano-electrical transducer channel, inhibited the Tl^+^ influx into the OHCs of the WT cochlea (n = 18 and 14 cochleae, respectively). ****p < 0.001 determined by Student's *t*-test. (**F, G**) ΔF_0-120_/F_0_ depended on the activity of Kv7.4 in the basolateral membrane of the OHCs, given that the Kv7.4 inhibitor XE991 terminated the Tl^+^ influx into the OHCs of WT cochlea (n = 8 and 8 cochleae, respectively). ****p < 0.001 determined using Student's *t*-test. (**H**) Changes in FluxOR fluorescent intensity were measured from 0 s (at the point of Tl^+^ addition) to 120 s in the cochlea of the WT, AAV-injected *Kcnq*4^W276S/+^, and non-injected *Kcnq*4^W276S/+^ mice. The yellow dotted line shows the boundary between the inner hair cells and OHCs. Scale bar = 50 μm. (**I**) F/F_0_ values after the Tl^+^ stimulus in the OHCs of the cochlea of the WT (n = 13 cochleae), AAV-injected *Kcnq*4^W276S/+^ (n = 18 cochleae), and non-injected *Kcnq*4^W276S/+^ mice (n = 27 cochleae) were measured. (**J**) ΔF_120-0_/F_0_ (from 0 to 120 s) for the OHCs of each group was compared. ****p < 0.001 by one-way ANOVA followed by Tukey's correction for multiple comparisons.

Thus, we hypothesized that the surviving cells after *Kcnq4* c.830G>C allele disruption functionally differ from the surviving cells with the mutant allele, given that *KCNQ4* p.W276S has a dominant-negative effect and the disruption of the mutant alleles enables WT subunits to assemble into functionally intact potassium channels. To examine this hypothesis, we developed a novel *ex vivo* hair cell thallium (Tl^+^) imaging technique that takes advantage of the well-described permeability of potassium channels to Tl^+^ ions 39 and the activity of potassium channels, to determine the membrane potential of the hair cells 40. The potassium (or Tl^+^) influx into hair cells via non-selective cationic channels is affected by the electrochemical gradient in the resting state, and thus, the amount of ion influx depends on the membrane potential of the OHCs. Consequently, more hyperpolarized cells are favorable for thallium ion uptake (**Figure 5A**). As Kv7.4 in the basolateral surface can hyperpolarize hair cells 17, we speculated that the restoration of Kv7.4 activity through the disruption of the mutant allele would result in a more hyperpolarized membrane potential in the OHCs, and subsequently increase the Tl^+^ ion influx. To evaluate the electrophysiological restoration of Kv7.4 by AAV injection, the uncapped cochlea incubated with the dye FluxOR, which is sensitive to thallium ion concentration (excitation/emission, 490/525 nm) 41, was visualized using fluorescence microscopy after a high concentration of Tl^+^ ions had been added onto the apical surface of the Corti organ (**Figure 5B**). We found that the Tl^+^ ion influx was prominent in OHCs of the *Kcnq4*^+/+^ mice (**Figure 5C** and **Movie S1**).

The intensity of FluxOR fluorescence (F) in the regions of the OHCs (representative of the OHC concentration of thallium ions) before the addition of Tl^+^ and after 120 s were then measured. Subsequently, we calculated the changes in fluorescence intensity (ΔF) relative to the baseline intensity (F_0_), as this indicates Tl^+^ influx into the OHCs. The ΔF/F_0_ increased with the addition of the Tl^+^ ions and its influx depended on the leaky activity of the mechano-electrical transduction (MET) channels during the resting state, given that quinone, the MET inhibitor, abolished the Tl^+^ influx into the OHCs of the WT cochlea (**Figures 5D-E**). As we did not use mechanical stimulation to activate the hair cells, thallium influx solely depended on their membrane potentials, regardless of the activation state of the MET (*i.e*., TMC1) 42-44. Furthermore, we found that the thallium ion influx depended on the activity of Kv7.4 in the basolateral membrane of the OHCs, as treatment with the Kv7.4 inhibitor XE991 terminated the Tl^+^ influx into the OHCs (**Figures 5F-G**). This led us to speculate that the Tl^+^ influx may be inhibited if Kv7.4 activity is decreased in the OHCs of the *Kcnq4*4^W276S/+^ mice.

Next, we measured the changes in F from 0 to 120 s after the addition of Tl^+^ to the cochlea of the WT, AAV-injected *Kcnq*^4W276S/+^, and non-injected *Kcnq*^4W276S/+^ mice. As results, the F values rarely changed in the OHCs of the non-injected *Kcnq*^4W276S/+^ when compared to those of the WT (**Figure 5H**). Notably, the intensity increased more rapidly in the OHCs of the AAV-injected mice than in those of the non-injected mice. AAV-injected OHCs showed a significantly increased thallium influx slope after the apical addition of Tl^+^ ions when compared to the non-injected OHCs (**Figure 5I-J**). These findings indicated that the surviving hair cells in *Kcnq4*^W276S/+^ mice after the mutant allele disruption by gene editing are more hyperpolarized than the non-injected *Kcnq*^4W276S/+^.

### Dominant-negative KCNQ4 variants linked to DFNA2 are suitable for SpCas9 gene editing

**Figure 6:**
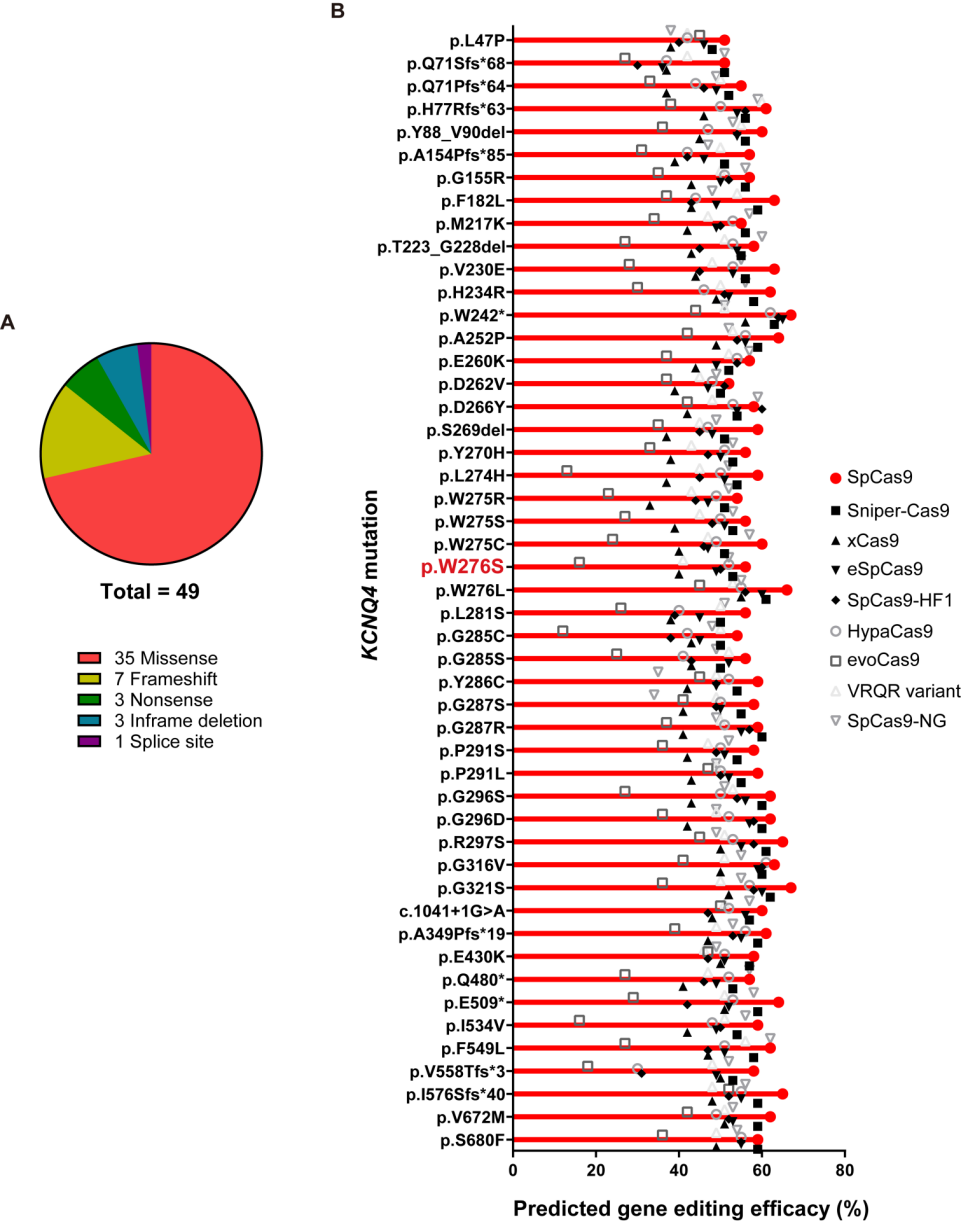
***In vivo* gene editing scores for variants in *KCNQ4***. (**A**) When pathogenic *KCNQ4* variants are classified by the variant type, missense variants are predominant, followed by frameshift variants. (**B**) In total, 49 variants linked to deafness non-syndromic autosomal dominant 2 (DFNA2) were selected for evaluating *in vivo* gene editing efficacies. Missense and frameshift variants, as well as splicing variants, were included. The DeepSpCas9 prediction program was utilized to test the predicted efficacy of *in vivo* gene editing based on combinations of candidate sgRNAs for *KCNQ4* variant sequences and Cas9 variant forms.

To evaluate the clinical feasibility of using *in vivo* gene editing to reverse the hearing loss caused by *KCNQ4* variants, we investigated all the DFNA2-related *KCNQ4* variants that have been reported in public databases, including the Human Gene Mutation Database (HGMD professional v2020.4) and ClinVar. As most of the variants linked to DFNA2 were found to be dominant-negative, the disruption of the mutant alleles using Cas9 is a feasible strategy to prevent hearing loss in DFNA2 18,22,45. We analyzed 49 variants in *KCNQ4*, including missense (*n* = 35), frameshift (*n* = 7), nonsense (*n* = 3), in-frame deletion (*n* = 3), and splice site (*n* = 1) variants (**Figure 6A**). To determine whether different Cas9 variants would be applicable for the specific destruction of the corresponding variants in *KCNQ4*, we predicted the gene editing scores for possible sgRNAs targeting each variant using the DeepSpCas9 prediction program (**Table S4**) 46. We found that the gene editing scores for most variants were comparable to or slightly higher than those for p.W276S, and WT SpCas9 presented the highest predicted efficacy among the Cas9 variants for all *KCNQ4* variants analyzed (**Figure 6B** and **Table S4**). Based on these findings, we concluded that most dominant-negative variants of KCNQ4 are potential targets for *in vivo* gene editing.

## Discussion

In the present study, we successfully applied *in vivo* gene editing with Cas9 nuclease to ameliorate progressive hearing loss in a dominant-negative *Kcnq4*^W276S/+^ murine model, although the improvement of auditory function is needed to be further advanced. Moreover, we showed that gene editing with SpCas9 targeting OHCs could restore the membrane potential of OHCs in the cochlea. However, functional recovery of OHCs via *Kcnq4* gene editing might not be the sole factor for hearing function improvement as other anatomic sites within inner ear might have been affected by gene editing.

While *in vivo* genome editing can remedy a range of genetic diseases, its cochlea-targeting capabilities need to be further improved. The delivery of gene editing materials to the cochlea is very difficult compared to delivery to organs, such as the liver, muscle, brain, and eye 47 as the cochlea is surrounded by a hard cortical bone and the blood-labyrinth barrier, which hinder efficient delivery. However, our results indicated that a high gene editing efficacy was not necessarily required to restore hearing in the *Kcnq4*^W276S/+^ murine model; specifically, a gene editing efficacy of approximately 0.6% at the genomic DNA level achieved with dual AAV plasmids of split SpCas9 and sgRNA partially rescued the auditory phenotype. Consistent herewith, a previous study reported sufficient hearing restoration in Beethoven mice harboring the *Tmc1* mutant allele with an *in vivo* gene editing efficacy of approximately 0.6% 48. Notably, the relatively low gene editing efficacy could lead to considerable hearing restoration, and this could be explained in several ways. For example, it is speculated that even a low gene editing efficacy could sufficiently (but not permanently) prevent progressive hearing loss if a small number of functional hair cells are stimulated.

Technically, following the preparation of the cochlear samples for deep sequencing, the actual number of disrupted mutant alleles may have been underestimated owing to contamination with other cell types in some cases. Nonetheless, the results may simply indicate that the gene editing efficacy threshold required for observable hearing restoration in the cochlea is lower than that for other diseases.

To date, various genome editing techniques have been used to treat human diseases 24. Gene editing strategies for combating diseases should be tailored to each pathogenetic mechanism. While gain-of-function or dominant-negative variants are potential targets for both pathogenic allele correction and disruption, loss-of-function or haploinsufficiency variants are a target only for allele correction. Given that most cases of congenial genetic deafness are associated with loss-of-function variants with an autosomal recessive inheritance pattern 49, allele-specific correction using base or prime editors could be considered a therapeutic strategy. For example, in the Baringo mouse model mimicking human recessive hearing loss (DFNB7/11), a cytosine base editor was found to correct the *Tmc1* mutant allele 30. Conversely, gain-of-function or dominant-negative variants are the main pathogenetic causes of autosomal dominant types of hearing loss, in which allele correction or allele disruption may be a therapeutic option. In a Beethoven mouse model mimicking human dominant hearing loss (DFNA36), the dominant-negative allele in *Tmc1* was successfully disrupted by the CRISPR/Cas9 nuclease 28,29. In this study, we specifically disrupted the dominant-negative allele in *Kncq4* in a mouse model mimicking DFNA2. However, allele correction strategies using base or prime editors could also be considered for dominant disease models with a single nucleotide variant. Nearly half of all pathogenic changes related to human diseases are single nucleotide variations 50. Specifically, 10% to 53% of the single nucleotide variations related with hearing loss have been estimated to be correctable using base or prime editors 25. Therefore, base or prime editors are a promising choice for the application of dominant-negative disease models. Nonetheless, the low efficacy and fidelity of these systems is currently a major translational hurdle.

Genetic alterations in *KCNQ4* result in the absence of potassium recycling in the OHCs of the cochlea, a phenomenon that enhances susceptibility to noise and promotes progressive hearing loss in DFNA2 10. In this aspect, genome editing to treat ARHL should focus on the OHCs, which are the sites where this sensory organ begins to degenerate. Notably, OHC degeneration occurs slowly over decades after onset of ARHL, suggesting that the therapeutic window for treating ARHL using gene therapy is adequate for attempting to decelerate progressive hearing loss 51,52. Given that most DFNA2-related variants, including indels, can be efficiently disrupted by SpCas9 and its variant forms (**Figure 6B**), we suggest that this gene editing strategy, *i.e*., inactivating *KCNQ4* dominant-negative mutant alleles, can be applied to other *KCNQ4* variants as well.

Although our data indicate that *in vivo* gene editing is applicable for the treatment of DFNA2, the gene editing efficacy and delivery vehicle should be further improved. In our study, OHC degeneration and absence of DPOAE were not able to be sufficiently prevented even after *in vivo* gene editing; subsequently, the improvement of auditory thresholds was partially observed. The partial restoration of hearing after genome editing in *Kcnq4*^W276S/+^ murine model may be attributable to the Kcnq4 expression in other inner ear regions or central auditory tract 53,54. Nevertheless, it is necessary to improve the viral transduction efficacy for *in vivo* gene editing in OHCs. For instance, the OHC transduction rate could be improved with the recently developed AAV9-PHP.B or AAV-S capsid 48,55,56, which can be used for *in vivo* gene editing in future studies. Moreover, other delivery methods, such as those based on mRNA and RNP, should be considered to avoid the collateral safety issues associated with administering virus injections to humans. RNP injections aid the silencing of dominant-negative alleles in the cochlea, as seen in the present and previous studies 28. However, this strategy is still not preferred in terms of clinical comfortability, because the administration of RNP via cochleostomy is a challenging procedure for many otology surgeons.

There are many issues that must be addressed prior to any clinical trial using genome editing technology for the treatment of deafness. First, safety concerns related to the application of gene editing in the cochlea should be sufficiently addressed. Off-target and unwanted long-lasting effects of Cas9 are the major drawbacks of the CRISPR/Cas9 system. In this study, the *in vitro* off-target effect of gRNA T3 was negligible; however, this needs to be validated further. To reduce the off-target effects of Cas9 nucleases, not only allele-specific sgRNAs, but also allele-specific PAM-detecting Cas9 nucleases should be designed and selected, as shown in a previous study 29. Second, higher gene editing efficacies for deafness are required before these strategies can be adopted in the clinic. Finally, the development of diverse animal models is a prerequisite for the broad application of gene editing in the treatment of deafness.

In conclusion, we demonstrated that the disruption of *Kcnq4* dominant-negative allele in OHCs in an effort to enhance the functional Kv7.4 channel activity partially restored hearing function in a murine model that recapitulated DFNA2, even at low gene editing efficacies. Our findings provide a rationale for the clinical application of gene editing for the treatment of ARHL.

## Methods

### Generation of the Kcnq4 p.W276S mouse model

Animal experimental protocols were reviewed and approved by the Institutional Animal Care and Use Committee of Yonsei University College of Medicine (#2017-0295). *Kcnq4* p.W276S knock-in mice were generated at Macrogen, Inc. (Seoul, Korea). Briefly, C57BL/6N female mice were treated with mare serum gonadotropin (7.5 IU) and human chorionic gonadotropin (5 IU). After 48 h, the mice were allowed to mate with C57BL/6N stud male mice. The next day, female mice with vaginal plugs were euthanized, and the fertilized embryos were harvested. A mixture of sgRNA (5′-CCTCCTATGCCGACTCGCTCTGG-3′), Cas9 protein, and ssDNA donor template (5′TCTACCTGGCTGAGAAGGATGCCAACTCTGACTTCTCCTCATATGCCGACTCGCTCTGGTCGGGGACGGTGCGTGAGCATCTGTGCAGGGCTGCCCTTACC-3′) was microinjected into one-cell embryos, which were then incubated at 37 °C for 1-2 h. The injected one-cell staged embryos (*n* = 14-16) were transplanted into the oviducts of pseudopregnant recipient mice (ICR). F0 mice were genotyped by PCR using tail cut samples (primers: 5′-GCATTCCTAGGGGTCTTTCC-3′; 5′-CATCAGGTTCTTGCGAACCT-3′) and subjected to a T7E1 assay. T7E1-positive samples were subjected to TA cloning and analyzed by Sanger sequencing. In the F1 generation, genotyping was performed using PCR with the same primers, and PCR products were digested using NdeI for 2-3 h to identify the two-band pattern in agarose gel electrophoresis.

### Plasmid construction

Cas9 and sgRNAs were expressed using the CMV promoter-driven Cas9-2A-mRFP-2A-Puro plasmid (hereafter, Cas9-puro vector) and the hU6 promoter-driven sgRNA plasmid, respectively. The plasmids were purchased from Toolgen (Seoul, Korea); vector maps of these plasmids are shown in **Figure S2B-C**.

For the AAV experiments, previously validated expression plasmids were used to express split intein-Cas9 plasmids (N-Cas9 N-intein and C-intein C-Cas9 plasmids, a gift from Oskar Ortiz) 57. To prepare sgRNA-N-Cas9-N-intein, we modified the backbone vector (Addgene plasmid #60958) generated by Swiech et al. 58. Briefly, the backbone vector was digested with XbaI and EcoRI, and an N-Cas9-N-intein fragment digested with XbaI and EcoRI was ligated into the backbone vector to express U6-sgRNA-N-Cas9-N-intein. Finally, to clone the selected sgRNA (sgRNA-T3), the vector was digested with SapI and ligated with annealed oligonucleotides. All plasmid and oligonucleotide sequences are provided in **Table S5** and **Note S1**.

### sgRNA preparation and reporter cell line transfection

The sgRNA target sequences were manually designed based on PAM (5′-NGG-3′) sequences near the variant target locus (c.830G>C) (**Figure S2C**). The sgRNA-encoding vector (pRG2-sgRNA) was digested with BsaI and ligated with the annealed oligonucleotides; the oligonucleotide sequences are listed in **Table S5**. Reporter cells for the *Kcnq4* mutant were transfected with plasmid mixtures containing Cas9-puro- and individual U6-sgRNA-encoding plasmids at a 1:2 weight ratio using Lipofectamine 2000 (Invitrogen, Carlsbad, CA, USA) according to the manufacturer's instructions and previously reported methods 59,60. The cells were harvested 3 days after transfection and analyzed.

The lenti-reporter plasmid constitutively expresses *RFP* and the *Kcnq4* target sequence containing a c.830G>C variant (35 bp in length), along with sequences encoding eGFP positioned out-of-frame relative to *RFP* (**Figures S2D**). The target sequence contains a 20-bp region with adjacent PAMs for gRNA binding and Cas9-mediated cleavage, resulting in double-strand breaks. The recruitment of Cas9 to the *Kcnq4* mutant-specific target sequence by properly spaced sgRNA results in a double-strand break, leading to an indel variant at the target locus. Because codons are triplets of nucleotides, one out of three repairs results in a significant “in-frame” fusion of *eGFP* with *mRFP* located upstream of the cleavage site.

### *In vitro* transcription sgRNA preparation

sgRNA was transcribed *in vitro* using T7 RNA polymerase with templates generated through annealing and extension of two complementary oligonucleotides (**Table S5**) using an mMESSAGE mMACHINE® T7 Ultra RNA Synthesis kit (#AM1345; Invitrogen) according to the manufacturer's instructions. RNA was purified using a MEGAclear kit (#AM1908; Invitrogen). Purified RNA was quantified using a Nanodrop system and gel electrophoresis.

### Lentivirus production and reporter cell line generation

Oligonucleotides, including the target sequence (**Table S5**), were synthesized at Macrogen Inc. and annealed *in vitro* using a thermocycler (95 °C for 5 min and then ramped down to 25 °C at 5 °C/min). The annealed oligonucleotides were ligated into the Lenti-reporter vectors digested with EcoR1 and BamH1.

The lentivirus was produced as previously described 35. Briefly, three transfer plasmids, containing the *Kcnq4* variant, psPAX2, and pMD2.G, were mixed at a weight ratio of 4:3:1 to yield a plasmid mixture (10 μg). HEK293T cells at 80%-90% confluence were transfected with the mixture using Lipofectamine 2000. The viral supernatant was harvested 48 and 72 h post-transfection, filtered through a Millex-HV 0.45-μm low-protein-binding membrane (Millipore, Darmstadt, Germany), and concentrated by ultracentrifugation.

Next, a reporter cell line was generated as previously described 35. Briefly, HEK293T cells (2.2 × 10^6^) were seeded in a 100-mm cell culture dish and incubated overnight. The cells were transduced with the lentiviral reporter at a multiplicity of infection of 0.1 with polybrene (4 μg/mL) and incubated for 15-18 h. Non-transduced cells were removed by zeocin (2 μg/mL; InvivoGen, Toulouse, France) treatment from day 3 to day 5. When nearly all non-transduced cells had died and the surviving cells had expanded, the cells were maintained in zeocin (2 μg/mL) for further use, as previously described 61. The reporter cell line expressing *Kcnq4* with the appropriate SpCas9 and sgRNA expression plasmids was used for transfection experiments. The activity of each sgRNA was detected at 72 h post transfection.

### MEF cell line generation

MEFs of Kcnq4 p.W276S mouse model were generated as previously reported 62. We used pregnant mice 14 to 16 days after the appearance of the copulation plug. After cervical dislocation outside the tissue culture hood, the uterus was removed by making an incision in the abdomen with micro scissors, lifting the uterine horn with forceps, and cutting with micro scissors. After removing the embryo from the uterine tissue, the blood was removed from each embryo by stirring using DPBS, while the upper eye and red tissue of the embryo were removed. The embryos from which the upper part of the eye and red tissue had been removed were transferred to 0.25% Trypsin-EDTA and chopped to a size of 1-2 mm with a sterile razor. Thereafter, resuspension and incubation at 37 °C for 10 min were repeated twice, following which the embryos were transferred into a 50 mL conical tube; resuspension was subsequently performed after adding MEF culture media. After 5 min, the supernatant was transferred to a T75 flask and incubated in a 37 °C incubator overnight. In subsequent passages, MEFs were used for the experiment.

### Off-target analysis

For analysis of the off-target effects in MEFs harboring the *Kcnq4* target mutant (c.830G>C) loci with selected sgRNA-T3, Cas-OFFinder was used to estimate the potential off-target genomic loci, and the corresponding primers for PCR were designed for the predicted top-ranking potential seven off-target sites, including *Kcnq4* WT loci (**Tables S3** and **S5**). Genomic DNA isolated after three days of post transfection from the MEFs harboring the *Kcnq4* target mutant allele (c.830G>C) with or without Cas9 treatment was used as the experimental group or negative control, respectively.

### Cell culture

HEK293T cells were purchased from the American Type Culture Collection (ATCC; Manassas, VA, USA). HEK293T cells, reporter cells, and Neuro2a cells were maintained in Dulbecco's modified Eagle's medium (Invitrogen) supplemented with 100 U/mL penicillin, 100 µg/mL streptomycin, and 10% fetal bovine serum.

### T7E1 assay

The T7E1 assay was performed as previously described 36. Briefly, the target site was amplified using nested PCR with appropriate primers (**Table S5**). Amplicons were denatured by heating and annealed to allow the formation of heteroduplex DNA and treated with T7 endonuclease 1 (5 U; New England Biolabs) at 37 °C for 20 min, followed by electrophoresis on a 2% agarose gel. Variant frequencies were calculated as previously described based on band intensities measured using ImageJ and using the following equation 63: variant frequency (%) = 100 × (1 - [1 - fraction cleaved]^1*/*2^), where the fraction cleaved is the total relative density of cleavage bands divided by the sum of the relative densities of the cleavage bands and uncut bands.

### DNA extraction and targeted deep sequencing

Genomic DNA extracted from HEK293T cells was purified using the Wizard Genomic DNA Purification Kit (Promega, Madison, WI, USA) according to the manufacturer's instructions. Mouse genomic DNA was extracted from surgically resected cochlear tissues using an Exgene Tissue SV Kit (GeneAll, Seoul, Korea). As isolated OHCs yielded low quantity DNA, the organ of Corti was isolated and analyzed. The on-target region within the genomic DNA was amplified using eTaq or Pfu DNA polymerase (Promega). Equal amounts of PCR amplicons were subjected to paired-end read sequencing using Illumina MiSeq at Bio Medical Laboratories (Minworth, UK). The region, including the target sites, was amplified by nested PCR using appropriate primers (**Table S5**). Indels mapped around the Cas9 nuclease cleavage site (3 bp upstream of PAM) were considered the result of nuclease-induced NHEJ-mediated mutagenesis.

### Allele-specific indel analysis

The gene editing efficacy of CRISPR for mutant alleles was analyzed using allele-specific indel analysis strategies. The gene editing efficacy for mutant alleles rather than WT alleles was prioritized. Because indels at the variant site can hinder the identification of the origin of the edited allele (*i.e*., whether the WT or mutant allele was edited), the mutant allele was identified using the synonymous variant c.810C>A located 20 bp upstream of the variant spot; we only counted reads with synonymous variants. Reads with both the synonymous variant and any out-of-frame indel pattern of 1 to 8 bp were regarded as edited mutant alleles, ruling out sequencing errors and in-frame indel variants. Furthermore, gene editing efficacies in injected and non-injected cochlea from the same mouse and uninjected control mice were compared to minimize inter-individual variation and background noise in sequencing analysis, respectively.

### AAV vector generation

Plasmids of split Cas9 (*i.e.*, C-Cas9 and N-Cas9) and gRNA were packaged into AAV/Anc80 using the Harvard viral core (in Boston Children Hospital) 38. AAV titers were validated using reverse transcription qPCR targeting the inverted terminal repeat of the virus (**Figure S4C**). AAV stock concentrations were 1.29 × 10^12^ and 4.18 × 10^12^ genome copies/mL for C-Cas9 and N-Cas9 with sgRNA, respectively. The final injected titer of combined AAV was estimated to be 1.00 × 10^9^ genome copies/µL in one cochlea.

### Optimization of RNP injection material using *in vitro* and explant samples

To determine an optimal injection ratio and incubation time for the RNP complex, we tested RNP mixture combinations at different ratios as shown in **Figure S11A-B**. Briefly, purified Cas9 proteins and sgRNAs at different ratios were incubated at 25 °C for 5 min, followed by complexation with a cationic lipid (Lipofectamine 2000) in OptiMEM (Life Technologies, Carlsbad, CA, USA) according to the manufacturer's protocol for DNA plasmid transfection. After a 20-min incubation, the sample mixture containing the cationic lipid and RNP was added to cells or injected into the cochlea. The optimal ratio was determined after comparing the indel percentages in four mixtures of Cas9, sgRNA, Lipofectamine 2000, and media as determined using deep sequencing. Although *in vivo* conditions are entirely different from those *in vitro* and in explant culture systems, the optimal injection mixture ratio may not markedly differ between *in vivo*, *ex vivo*, and *in vitro* conditions. The *ex vivo* gene editing efficacy varied among the different combinations (from 26.1% to 32.2%; **Figure S11B**). As the physiologically available volume for injection into the cochlea of P2-P5 pups is limited to 1 µL, the sgRNA (1.0 µg) and Cas9 (1.5 µg) concentrations were maximized.

### Inner ear injection

AAV virus or RNP complex were injected into the inner ear of *Kcnq4* p.W276S heterozygous mutant pups at P1-P3. After the pups were anesthetized by exposure to ice for 2 min (hypothermia), a post-auricular incision was made to expose the injection route. Using a glass pipette and a Nanoliter2020 Injector (World Precision Instruments, Hertfordshire, UK), the injection material (1 µL) was delivered into the cochlea at a constant rate of 40 nL/min. After injection, a suture was made to close the cut skin. The pup was then placed on a heating pad to recover for at least 5 min. Various injection routes were comprehensively tested in more than 500 pups to minimize physical damage to the cochlea during injection.

### ABR and DPOAE hearing tests

ABR thresholds were measured in a sound-proof chamber using the Tucker-Davis Technologies (TDT) RZ6 digital signal processing hardware and BioSigRZ software (Alachua, FL, USA). Sub-dermal needles (electrodes) were positioned at the vertex and ventrolateral to the right and left ears of anesthetized mice. Calibrated click stimuli (duration: 10 µs) or tone burst stimuli (duration: 5 ms) were produced at 6, 12, 18, 24, and 30 kHz using the SigGenRZ software and the RZ6 digital signal processor and were delivered into the ear canal using a multi-field 1 (MF1) magnetic speaker (TDT). The stimulus intensity was increased from 10 to 90 dB SPL in 5-dB steps. The ABR signals were fed into a low-impedance Medusa Biological Amplifier System (RA4LI, TDT), which delivered the signal to the RZ6 digital signal processing hardware. The recorded signals were filtered using a 0.5-1-kHz band-pass filter, and ABR waveforms in response to 256 tone bursts were averaged.

For DPOAE, a combined TDT microphone-speaker system was utilized. Primary stimulus tones were produced using an RZ6 digital signal processor with the SigGenRZ software and were delivered using a custom probe with an ER 10B+ microphone (Etymotic, Elk Grove Village, IL, USA) and MF1 speaker positioned in the ear canal. Primary tones were set at a frequency ratio (f2/f1) of 1.2 with target frequencies of 6, 12, 16, 18, 22, 24, and 30 kHz. The f2 intensity levels were the same as the f1 intensity levels (L1 = L2). Sounds caused by the primary tones were received by the ER 10B+ microphone and recorded using the RZ6 digital signal processor. The DPOAE input/output (I/O) functions were determined at specific frequencies (6 and 30 kHz) with a frequency ratio (f2/f1) of 1.2 and equal intensity levels (L1 = L2). Intensity levels of the primary tones were increased from 20 to 80 dB SPL in 5-dB SPL increments. Fast Fourier transform (FFT) was performed for each primary tone for the DP-grams and at each intensity for the I/O functions using BioSigRZ to determine the average spectra of the two primaries, the 2f1-f2 distortion products, and the noise floors.

### Immunohistochemistry and histology

Injected and non-injected cochleae were excised after CO_2_ inhalation-induced euthanasia. Temporal bones were fixed overnight in 4% paraformaldehyde at 4 °C and decalcified in 120 mM EDTA for at least 1 week. The cochleae were dissected in pieces from the decalcified tissue for whole-mount immunofluorescence. Tissues were permeabilized with 0.3% Triton X-100, blocked with 10% donkey serum for 1 h, and then incubated at 4 °C overnight with the following primary antibodies: mouse anti-Tuj1, purified anti-tubulin beta 3, (1:300; 801202; BioLegend, San Diego, CA, USA), chicken anti-neurofilament H antibodies (1:1,000; AB5539; Merck Millipore), and rabbit anti-Myosin VIIa antibody (1:200; 25-6790, Proteus Biosciences). DAPI (1:5,000; Invitrogen) was used for nuclear staining (25 °C for 10 min). To count IHCs and OHCs, FITC-conjugated phalloidin (1:200; P5282; Sigma-Aldrich) was used to stain F-actin at 25 °C for 15 min. After three rinses with 0.3% Triton X-100 in 1× PBS, the specimens were incubated for 1 h with the secondary antibodies donkey anti-mouse Alexa Fluor 488 (A21202; 1:1,000; Invitrogen) and goat anti-chicken Alexa Fluor 568 (A11041; 1:1,000; Invitrogen), and donkey anti-rabbit Alexa Fluor 568 (1:1,000; A10042; Invitrogen). The specimens were mounted in Fluoromount^TM^ Aqueous Mounting Medium (F4680-25ML, Sigma-Aldrich) and imaged with a confocal microscope (LSM700; Zeiss, Jena, Germany) using a 10×, 20×, or 40× water-immersion lens with or without digital zoom. The images were processed using the ZEN software. To count IHCs, OHCs, and DAPI-positive and Tuj1-positive spiral ganglion neurons (SGN), to quantify the density of the neurofilament heavy chains, and to investigate the transduction efficiency of the Anc80-GFP, *z*-stacks were acquired using the maximum intensity projection method for each segment, in ImageJ. Composite images showing the entire cochlea were constructed in Adobe Photoshop CS3 to display the entire turn of the cochlea. A frequency map of each specimen was drawn using ImageJ. Phalloidin- and DAPI-positive IHCs and OHCs were counted in cochlear regions responsive to different sound frequencies, and segments containing dissection-related damage were omitted from the analysis. To count SGN, the fluorescence intensity of the neurofilament heavy chains was quantified at the inner spiral plexus (spiraling underneath the IHCs) and non-myelinated outer spiral fibers (spiraling underneath the OHCs) using the auto-threshold algorithm.

### Live imaging and thallium assay of cochlea

A thallium flux assay was performed *ex vivo* to confirm potassium-induced physiological changes in the hair cells of AAV-injected and non-injected cochlea. Briefly, 7-week-old mice with confirmed ABR-induced hearing improvement were euthanized with CO_2_ to harvest both cochlea (*i.e.*, AAV-injected and contralateral non-injected inner ears). Inner ears of age-matched WT mice were used as control. The apical bone capsule of the inner ear was removed in cold Hanks' balanced salt solution using forceps. Each cochlea extracted from the inner ear was fixed with a needle on a confocal glass bottom dish (100350; SPL). The FluxOR^TM^ Potassium Ion Channel Assay (F10016; Invitrogen) was performed according to the manufacturer's instructions. The cochleae were incubated *ex vivo* with FluxOR^TM^, a fluorescent dye (excitation wavelength, 488 mm) sensitive exclusively to thallium ions, for 1 h 41. To confirm whether the FluxOR dye loaded inside hair cells binds to extracellular Tl^+^ from the MET channel and increases the fluorescence density, WT cochlea treated with quinine (10 mM; a MET channel inhibitor) were used as negative control 64. After loading the FluxOR dye, it was replaced with assay buffer (100 μL), and live-cell imaging was conducted using a confocal microscope and the MetaFluor software. When the baseline was stabilized, a stimulus buffer (20 μL, 2 mM Tl^+^) was added, and recording was performed for 120 s. After adding the stimulus buffer, △F_120-0_ was calculated relative to the baseline. Range of interest was selected in the outer hair cell area, and the images at 0 and 120 s were analyzed.

### KCNQ4 variant collection and gene editing efficacy prediction

To collect all reported *KCNQ4* variants, we identified all *KCNQ4* variants annotated as “pathogenic/likely pathogenic” in the ClinVar database or as “disease-causing” in the HGMD database (professional v.2020.4). For a total of 49 pathogenic variants in *KCNQ4*, different combinations of sgRNAs and Cas9 variants were tested for gene editing efficacy in mutant alleles using our previously reported protocol 34. The best gene editing efficacy for each variant was compared across all variants and Cas9 variant types.

### Statistical analysis

Data were pooled from at least three independent experiments and are expressed as the mean ± SD. Means of two groups were compared using Student's *t*-test. To compare means of three or more than three groups, we used two-way analysis of variance (ANOVA) and Kruskal-Wallis test for parametric and non-parametric data, respectively. *p* < 0.05 was considered significant. All analyses were conducted using GraphPad Prism v8.0 (GraphPad Software, San Diego, CA, USA).

### Data and software availability

The data and codes associated with this study are available from the corresponding author upon reasonable request. The published article includes all datasets/codes generated or analyzed during this study. Deep sequencing data have been deposited in the NCBI Sequence Read Archive (PRJNA691110).

## Supplementary Material

Supplementary figures, movie heading, and note.

Supplementary tables.

Supplementary movie.

## Abbreviations

AAV: adeno-associated virus
ARHL: age-related hearing loss
IHC: inner hair cell
Indel: insertion and deletion
MEF: mouse embryo fibroblast
OHC: outer hair cell
sgRNA: single-guide RNA
WT: wild-type

## References

[1] Morton CC, Nance WE (2006). Newborn hearing screening-a silent revolution. N Engl J Med.

[2] WHO. Deafness and hearing loss. 2021.

[3] Goman AM, Reed NS, Lin FR (2017). Addressing Estimated Hearing Loss in Adults in 2060. JAMA Otolaryngol Head Neck Surg.

[4] Cunningham LL, Tucci DL (2017). Hearing Loss in Adults. N Engl J Med.

[5] Petit C, El-Amraoui A, Avan P Audition: Hearing and Deafness. 2016.

[6] Bowl MR, Dawson SJ (2019). Age-Related Hearing Loss. Cold Spring Harb Perspect Med.

[7] Yamasoba T, Lin FR, Someya S, Kashio A, Sakamoto T, Kondo K (2013). Current concepts in age-related hearing loss: epidemiology and mechanistic pathways. Hear Res.

[8] Kujawa SG, Liberman MC (2006). Acceleration of age-related hearing loss by early noise exposure: evidence of a misspent youth. J Neurosci.

[9] Van Laer L, Carlsson PI, Ottschytsch N (2006). The contribution of genes involved in potassium-recycling in the inner ear to noise-induced hearing loss. Hum Mutat.

[10] Rim JH, Choi JY, Jung J, Gee HY (2021). Activation of KCNQ4 as a Therapeutic Strategy to Treat Hearing Loss. Int J Mol Sci.

[11] Peixoto Pinheiro B, Vona B, Lowenheim H, Ruttiger L, Knipper M, Adel Y (2021). Age-related hearing loss pertaining to potassium ion channels in the cochlea and auditory pathway. Pflugers Arch.

[12] Wu PZ, O'Malley JT, de Gruttola V, Liberman MC (2020). Age-Related Hearing Loss Is Dominated by Damage to Inner Ear Sensory Cells, Not the Cellular Battery That Powers Them. J Neurosci.

[13] Bielefeld EC, Coling D, Chen GD (2008). Age-related hearing loss in the Fischer 344/NHsd rat substrain. Hear Res.

[14] Popelar J, Groh D, Pelanova J, Canlon B, Syka J (2006). Age-related changes in cochlear and brainstem auditory functions in Fischer 344 rats. Neurobiol Aging.

[15] Vaden KI Jr, Matthews LJ, Dubno JR (2018). Transient-Evoked Otoacoustic Emissions Reflect Audiometric Patterns of Age-Related Hearing Loss. Trends Hear.

[16] Ueberfuhr MA, Fehlberg H, Goodman SS, Withnell RH (2016). A DPOAE assessment of outer hair cell integrity in ears with age-related hearing loss. Hear Res.

[17] Kubisch C, Schroeder BC, Friedrich T (1999). KCNQ4, a novel potassium channel expressed in sensory outer hair cells, is mutated in dominant deafness. Cell.

[18] Kharkovets T, Dedek K, Maier H (2006). Mice with altered KCNQ4 K+ channels implicate sensory outer hair cells in human progressive deafness. EMBO J.

[19] Wangemann P (2002). K+ cycling and the endocochlear potential. Hear Res.

[20] Hibino H, Kurachi Y (2006). Molecular and physiological bases of the K+ circulation in the mammalian inner ear. Physiology (Bethesda).

[21] Coucke PJ, Van Hauwe P, Kelley PM (1999). Mutations in the KCNQ4 gene are responsible for autosomal dominant deafness in four DFNA2 families. Hum Mol Genet.

[22] Dominguez LM, Dodson KM (2012). Genetics of hearing loss: focus on DFNA2. Appl Clin Genet.

[23] Kamada F, Kure S, Kudo T (2006). A novel KCNQ4 one-base deletion in a large pedigree with hearing loss: implication for the genotype-phenotype correlation. J Hum Genet.

[24] Anzalone AV, Koblan LW, Liu DR (2020). Genome editing with CRISPR-Cas nucleases, base editors, transposases and prime editors. Nat Biotechnol.

[25] Niggemann P, Gyorgy B, Chen ZY (2020). Genome and base editing for genetic hearing loss. Hear Res.

[26] Ding N, Lee S, Lieber-Kotz M, Yang J, Gao X (2021). Advances in genome editing for genetic hearing loss. Adv Drug Deliv Rev.

[27] Géléoc GGS, El-Amraoui A (2020). Disease mechanisms and gene therapy for Usher syndrome. Hear Res.

[28] Gao X, Tao Y, Lamas V (2018). Treatment of autosomal dominant hearing loss by *in vivo* delivery of genome editing agents. Nature.

[29] Gyorgy B, Nist-Lund C, Pan B (2019). Allele-specific gene editing prevents deafness in a model of dominant progressive hearing loss. Nat Med.

[30] Yeh W-H, Shubina-Oleinik O, Levy JM (2020). *In vivo* base editing restores sensory transduction and transiently improves auditory function in a mouse model of recessive deafness. Sci Transl Med.

[31] Van Eyken E, Van Laer L, Fransen E (2006). KCNQ4: a gene for age-related hearing impairment?. Hum Mutat.

[32] Akita J, Abe S, Shinkawa H, Kimberling WJ, Usami S (2001). Clinical and genetic features of nonsyndromic autosomal dominant sensorineural hearing loss: KCNQ4 is a gene responsible in Japanese. J Hum Genet.

[33] Van Camp G, Coucke PJ, Akita J (2002). A mutational hot spot in the KCNQ4 gene responsible for autosomal dominant hearing impairment. Hum Mutat.

[34] Kim HK, Kim Y, Lee S (2019). SpCas9 activity prediction by DeepSpCas9, a deep learning-based model with high generalization performance. Sci Adv.

[35] Kim HK, Song M, Lee J (2017). *In vivo* high-throughput profiling of CRISPR-Cpf1 activity. Nat Methods.

[36] Kim YH, Ramakrishna S, Kim H, Kim JS (2014). Enrichment of cells with TALEN-induced mutations using surrogate reporters. Methods.

[37] Bae S, Park J, Kim J-S (2014). Cas-OFFinder: a fast and versatile algorithm that searches for potential off-target sites of Cas9 RNA-guided endonucleases. Bioinformatics.

[38] Landegger LD, Pan B, Askew C (2017). A synthetic AAV vector enables safe and efficient gene transfer to the mammalian inner ear. Nat Biotechnol.

[39] Geng Y, Hoke A, Delpire E (2009). The Ste20 kinases Ste20-related proline-alanine-rich kinase and oxidative-stress response 1 regulate NKCC1 function in sensory neurons. J Biol Chem.

[40] Fettiplace R, Fuchs PA (1999). Mechanisms of hair cell tuning. Annu Rev Physiol.

[41] Weaver CD, Harden D, Dworetzky SI, Robertson B, Knox RJ (2004). A thallium-sensitive, fluorescence-based assay for detecting and characterizing potassium channel modulators in mammalian cells. J Biomol Screen.

[42] Liu S, Wang S, Zou L (2019). TMC1 is an essential component of a leak channel that modulates tonotopy and excitability of auditory hair cells in mice. Elife.

[43] Corey DP, Holt JR (2016). Are TMCs the Mechanotransduction Channels of Vertebrate Hair Cells?. J Neurosci.

[44] Housley GD, Ashmore JF (1992). Ionic currents of outer hair cells isolated from the guinea-pig cochlea. J Physiol.

[45] Jung J, Choi HB, Koh YI (2018). Whole-exome sequencing identifies two novel mutations in KCNQ4 in individuals with nonsyndromic hearing loss. Sci Rep.

[46] Kim HK, Min S, Song M (2018). Deep learning improves prediction of CRISPR-Cpf1 guide RNA activity. Nat Biotechnol.

[47] van Haasteren J, Li J, Scheideler OJ, Murthy N, Schaffer DV (2020). The delivery challenge: fulfilling the promise of therapeutic genome editing. Nat Biotechnol.

[48] Wu J, Solanes P, Nist-Lund C (2021). Single and Dual Vector Gene Therapy with AAV9-PHP.B Rescues Hearing in Tmc1 Mutant Mice. Mol Ther.

[49] Jung J, Lee JS, Cho KJ (2017). Genetic Predisposition to Sporadic Congenital Hearing Loss in a Pediatric Population. Sci Rep.

[50] Anzalone AV, Randolph PB, Davis JR (2019). Search-and-replace genome editing without double-strand breaks or donor DNA. Nature.

[51] Jung J, Lin H, Koh YI (2019). Rare KCNQ4 variants found in public databases underlie impaired channel activity that may contribute to hearing impairment. Exp Mol Med.

[52] Shin DH, Jung J, Koh YI (2019). A recurrent mutation in KCNQ4 in Korean families with nonsyndromic hearing loss and rescue of the channel activity by KCNQ activators. Hum Mutat.

[53] Kharkovets T, Hardelin J-P, Safieddine S (2000). KCNQ4, a K+ channel mutated in a form of dominant deafness, is expressed in the inner ear and the central auditory pathway. Proceedings of the National Academy of Sciences.

[54] Beisel KW, Rocha-Sanchez SM, Morris KA (2005). Differential expression of KCNQ4 in inner hair cells and sensory neurons is the basis of progressive high-frequency hearing loss. J Neurosci.

[55] György B, Meijer EJ, Ivanchenko MV (2019). Gene transfer with AAV9-PHP. B rescues hearing in a mouse model of Usher syndrome 3A and transduces hair cells in a non-human primate. Molecular Therapy-Methods & Clinical Development.

[56] Ivanchenko MV, Hanlon KS, Hathaway DM (2021). AAV-S: A versatile capsid variant for transduction of mouse and primate inner ear. Molecular Therapy-Methods & Clinical Development.

[57] Truong DJ, Kühner K, Kühn R (2015). Development of an intein-mediated split-Cas9 system for gene therapy. Nucleic Acids Res.

[58] Swiech L, Heidenreich M, Banerjee A (2015). *In vivo* interrogation of gene function in the mammalian brain using CRISPR-Cas9. Nat Biotechnol.

[59] Lim JS, Gopalappa R, Kim SH (2017). Somatic Mutations in TSC1 and TSC2 Cause Focal Cortical Dysplasia. Am J Hum Genet.

[60] Gopalappa R, Suresh B, Ramakrishna S, Kim HH (2018). Paired D10A Cas9 nickases are sometimes more efficient than individual nucleases for gene disruption. Nucleic Acids Res.

[61] Shalem O, Sanjana NE, Hartenian E (2014). Genome-scale CRISPR-Cas9 knockout screening in human cells. Science.

[62] Shapiro J, Iancu O, Jacobi AM (2020). Increasing CRISPR Efficiency and Measuring Its Specificity in HSPCs Using a Clinically Relevant System. Mol Ther Methods Clin Dev.

[63] Guschin DY, Waite AJ, Katibah GE, Miller JC, Holmes MC, Rebar EJ (2010). A rapid and general assay for monitoring endogenous gene modification. Methods Mol Biol.

[64] Alharazneh A, Luk L, Huth M (2011). Functional hair cell mechanotransducer channels are required for aminoglycoside ototoxicity. PLoS One.

