# Supplementary material for: In vivo outer hair cell gene editing ameliorates progressive hearing loss in dominant-negative Kcnq4 murine model
Source: Theranostics. 2022 Feb 28;12(5):2465–82. doi: 10.7150/thno.67781 (PMC8899569; doi:10.7150/thno.67781)
Supplement: Supplementary file 1 — Supplementary figures, movie heading, and note. [file thnov12p2465s1.pdf]

Supplemental Information for

In vivo outer hair cell gene editing ameliorates progressive hearing loss in dominant-negative *Kcnq4* murine model

Byung Wha Noh, John Hoon Rim, Ramu Gopalappa, Haiyue Lin, Kyu Min Kim, Min Jin Kang, Heon Yung Gee*, Jae Young Choi*, Hyongbum Henry Kim*, Jinsei Jung*

*Corresponding authors: Email addresses: jsjung@yuhs.ac (Jinsei Jung); hkim1@yuhs.ac (Hyongbum Henry Kim); jychoi@yuhs.ac (Jae Young Choi); hygee@yuhs.ac (Heon Yung Gee)

This file includes:

Figure S1. Morphology of stereocilia in *Kcnq4*$^{W276S/+}$ mice and Kcnq4 expression in the cochlea of wild-type mouse.
Figure S2. Candidate sgRNAs targeting the *Kcnq4* mutant allele.
Figure S3. Optimization of sgRNA for *in vivo* gene editing.
Figure S4. Dual split AAV system for SpCas9 and sgRNA.
Figure S5. Optimization of delivery routes for *in vivo* gene editing.
Figure S6. Transfection of SpCas9 in the cochlea hair cells.
Figure S7. Safety of AAV and RNP injection into the scala media.
Figure S8. Characteristics of auditory brainstem responses (ABRs) in *Kcnq4*$^{W276S/+}$ after *in vivo* gene editing by AAV injection.
Figure S9. sgRNA-dependent hearing restoration by gene editing in *Kcnq4*$^{W276S/+}$ mouse.
Figure S10. Long-term effect of *in vivo* gene editing in *Kcnq4*$^{W276S/+}$.
Figure S11. Optimization of ribonucleotide complex (RNP) vehicles and *in vivo* phenotypic rescue.
Figure S12. Effect of *in vivo* gene editing on the hair cells and neurofilaments of *Kcnq4*$^{W276S/+}$ mice.
Figure S13. Effect of *in vivo* gene editing on neuronal survival and hair cell morphology in *Kcnq4*$^{W276S/+}$ mice.
Note S1. Coding sequences of the dual AAV plasmid system.
Caption for Movie S1. *Ex vivo* imaging of outer hair cells using thallium-sensitive dye (FluxOR-Tl^{+}) in the apical turn (6 kHz) of a wild-type cochlea.

Supplemental Figures

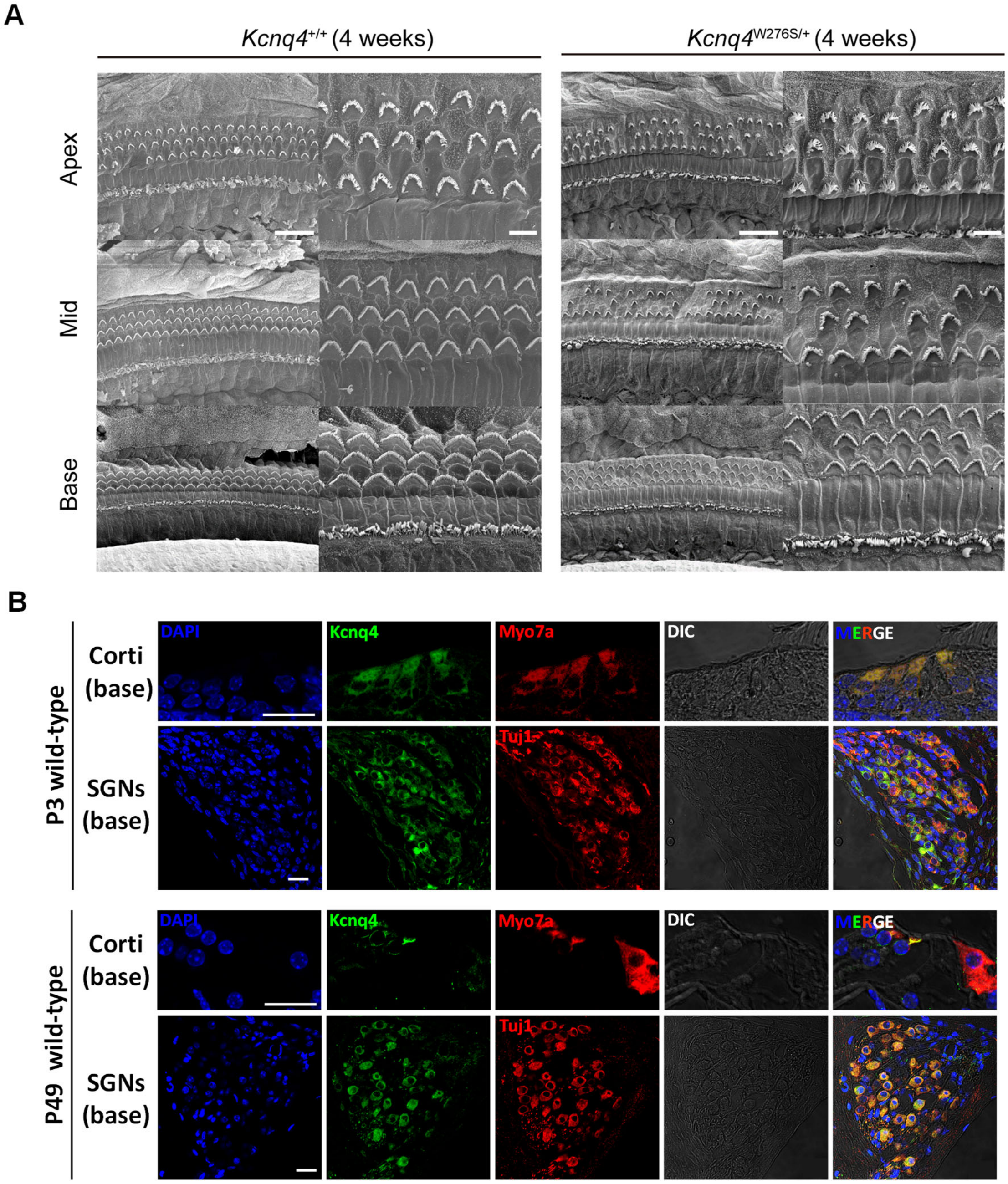

Figure S1. Morphology of stereocilia in *Kcnq4*$^{W276S/+}$ mice and Kcnq4 expression in the cochlea of wild-type mouse.
(A) Scanning electron micrographs of the apex, mid, and base regions of cochlea of 4-week-old wild-type (WT) and heterozygote mutant mice. Scale bars, 20 µm (low power) and 5 µm (high power). **(B)** Immunostaining of Kcnq4 was performed in the basal cochlea of P3 and P49 wild-type mice. Anti-DAPI (blue), anti-Kcnq4 (green), anti-Myo7a (red), and anti-Tju1 (red) antibodies were used. Corti, organ of the Corti; SGN, spiral ganglion; Scale bar, 20 µm.

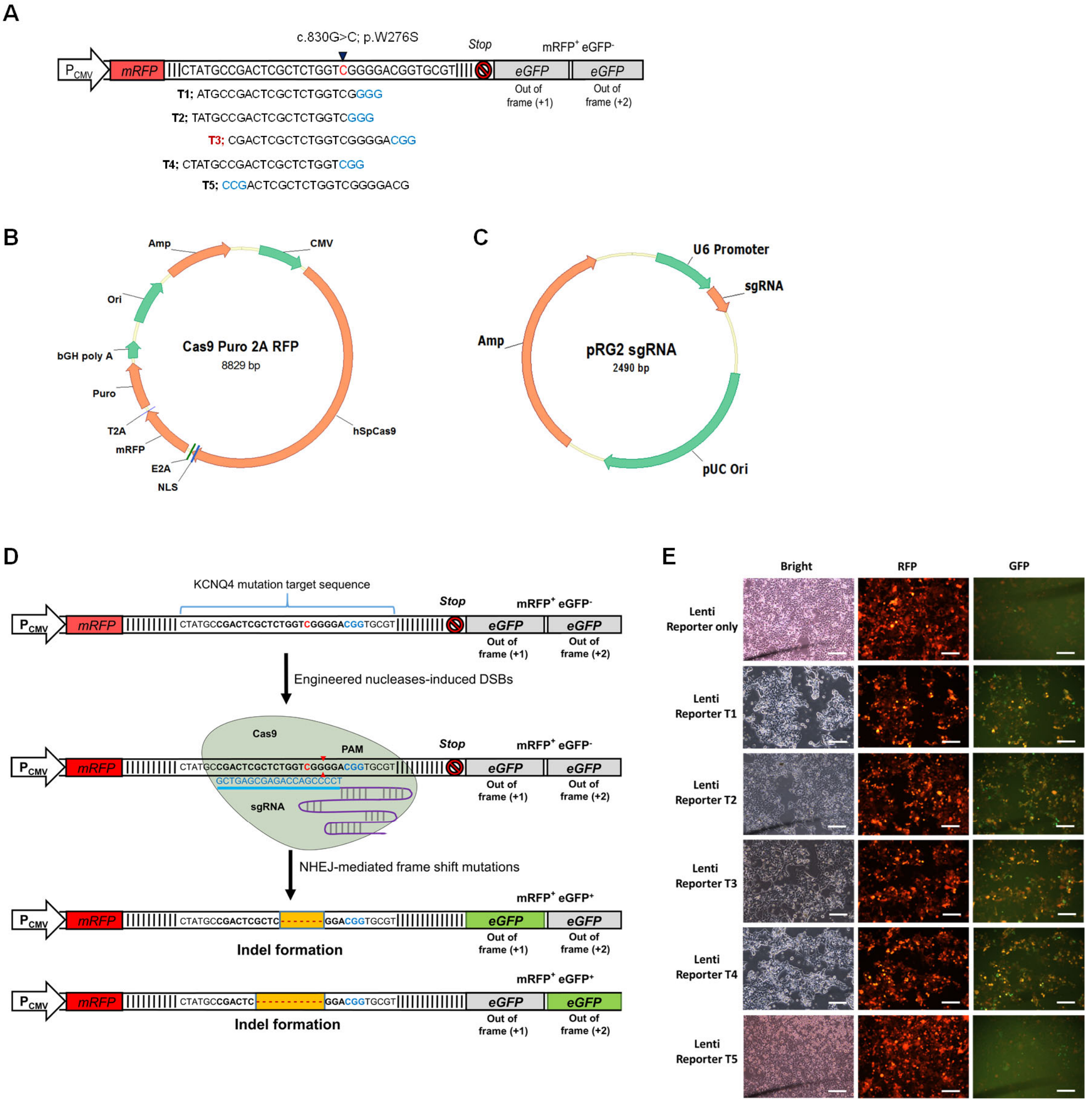

Figure S2. Candidate sgRNAs targeting the *Kcnq4* mutant allele.

(**A**) Schematic representation of the reporter construct for sgRNA selection. The protospacer-adjacent motif (PAM) sequence in each sgRNA is indicated in blue. (**B and C**) Vector maps of Cas9-2A-mRFP-2A-Puro (B) and pRG2-sgRNA (C). hSpCas9 (human codon-optimized Cas9 nuclease derived from *Streptococcus pyogenes*) is expressed by the CMV promoter, and the sgRNA is transcribed by the U6 promoter (pU6). The ampicillin resistance gene (*Amp*) enables the selection of transformed bacterial cells. E2A, equine rhinitis A virus (ERAV) 2A; mRFP, monomeric red fluorescent protein; T2A, *Thosea asigna* virus 2A; *Puro*, puromycin resistance gene; ITR, inverted terminal repeat; NLS, nuclear localization signal. (**D**) Mechanism of the fluorescent reporter system used in the sgRNA candidate selection process. The reporter plasmid constitutively expresses RFP and the target sequence encompassing the *Kcnq4* variant site (c.830G>C) (35 bp in length; mutant nucleotide in red) by the CMV promoter (P_{CMV}), whereas eGFP is not expressed unless Cas9 is active because the *eGFP* sequence is out-of-frame. If a double-strand break is introduced into the target sequence by the sgRNA (blue sequence) and Cas9 (light green), the double-strand break is repaired by error-prone nonhomologous end joining, which often results in an insertion/deletion (indel) variant. This variant causes frameshifts (orange bars with red dashes), leading to *eGFP* expression visualized by green fluorescence. The predicted Cas9-gRNA cutting position is indicated with red bars after the arrowhead. (**E**) Fluorescence micrographs of the reporter cell lines after transfection with the plasmid encoding Cas9 and each sgRNA targeting the *Kcnq4* variant target region. Scale bar, 50 µm.

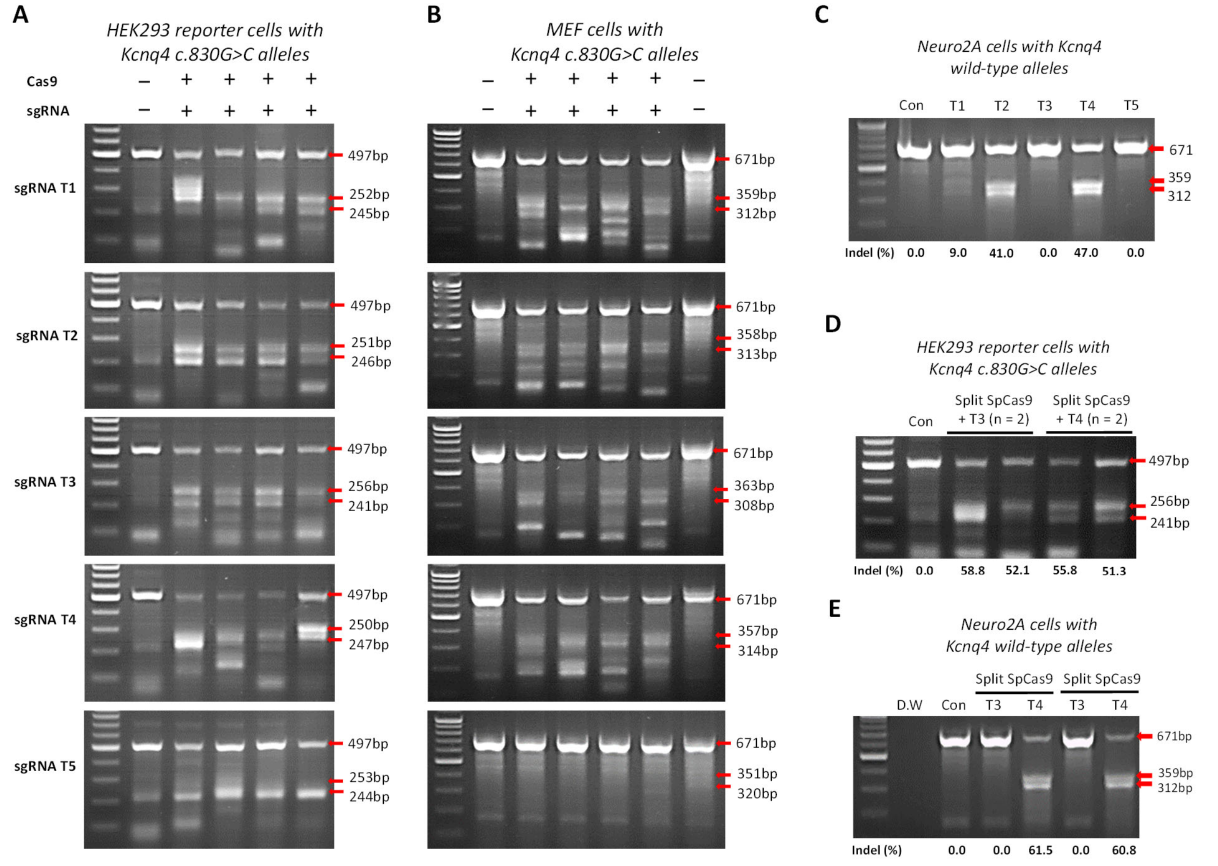

Figure S3. Optimization of sgRNA for *in vivo* gene editing.

(**A-B**) T7E1 assay results of on-target efficacy of sgRNA candidates determined in HEK293 reporter cells (A) and MEFs (B) harboring the *Kcnq4* target mutant allele (c.830G>C). The top band indicated as uncut DNA; the two smaller cleaved bands correspond to edited DNA (n = 4 in each gel) (**C**) Off-target activity for sgRNA candidates was evaluated in Neuro2a cells with *Kcnq4* wild-type alleles using T7E1 assay. The top band indicated corresponds to uncut DNA; the two smaller cleaved bands correspond to edited DNA. (**D**) On-target efficacy of split SpCas9 with T3 or T4 was evaluated by T7E1 assay in HEK293 reporter cells with *Kcnq4* c.830G>C allele. The top band indicated corresponds to uncut DNA; the two smaller cleaved bands correspond to edited DNA. con, control. **(E)** Off-target effect of split SpCas9 was compared to that of full SpCas9 in Neuro2a cells with *Kcnq4* wild-type alleles by T7E1 assay. The top band indicated corresponds to uncut DNA; the two smaller cleaved bands correspond to edited DNA. D.W., distilled water; con, control.

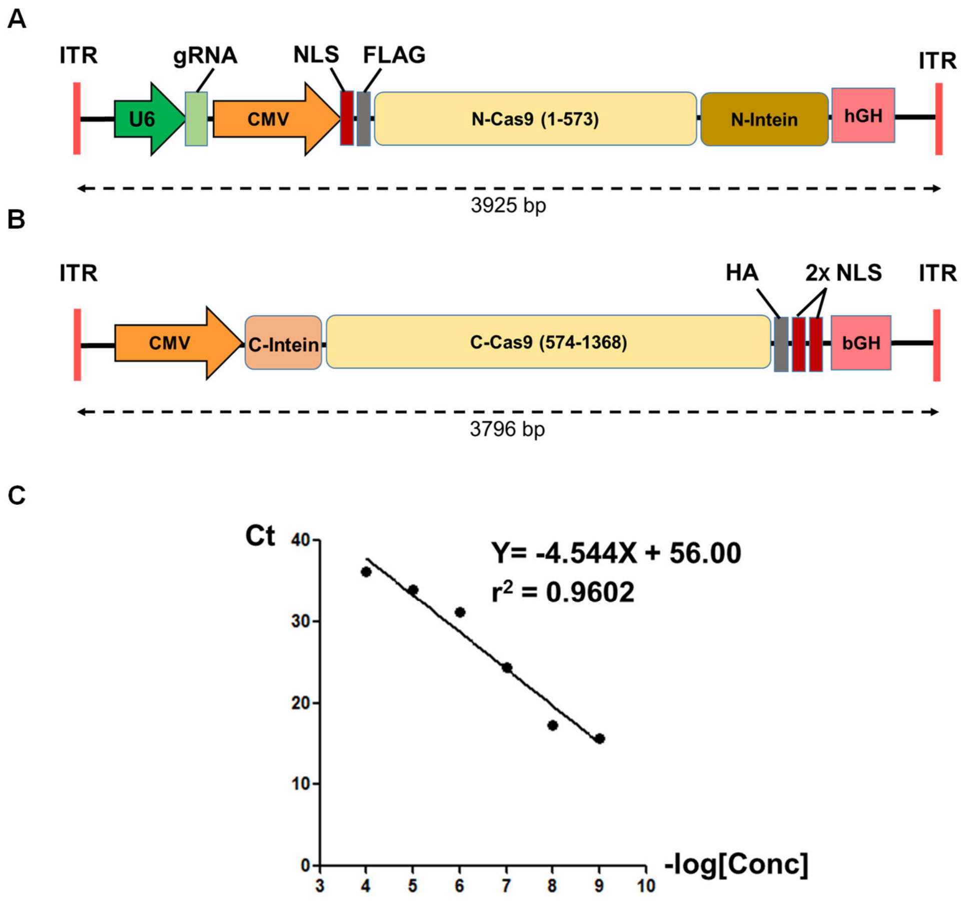

Figure S4. Dual split AAV system for SpCas9 and sgRNA.
(**A and B**) Overview of the split-SpCas9 expression plasmids U6-sgRNA-split–N-Cas9-N-intein (A) and C-intein-split–C-Cas9 (B). The U6 promoter was chosen (dark green) to express gRNA (light green). To ensure high expression, a strong synthetic mammalian promoter (CMV, orange) and a bovine growth hormone (bGH, red) polyadenylation site were used. Cas9 is shown in yellow, N-intein in dark brown, and C-Intein in light brown. FLAG and HA tags are shown in light and dark gray, respectively. Sequences of all plasmids used are listed in Supplemental Note 1. (**C**) Standard curve for the quantitation of split-SpCas9 encapsidated by Anc80L65 capsids. The viral titer was confirmed using quantitative reverse transcription PCR.

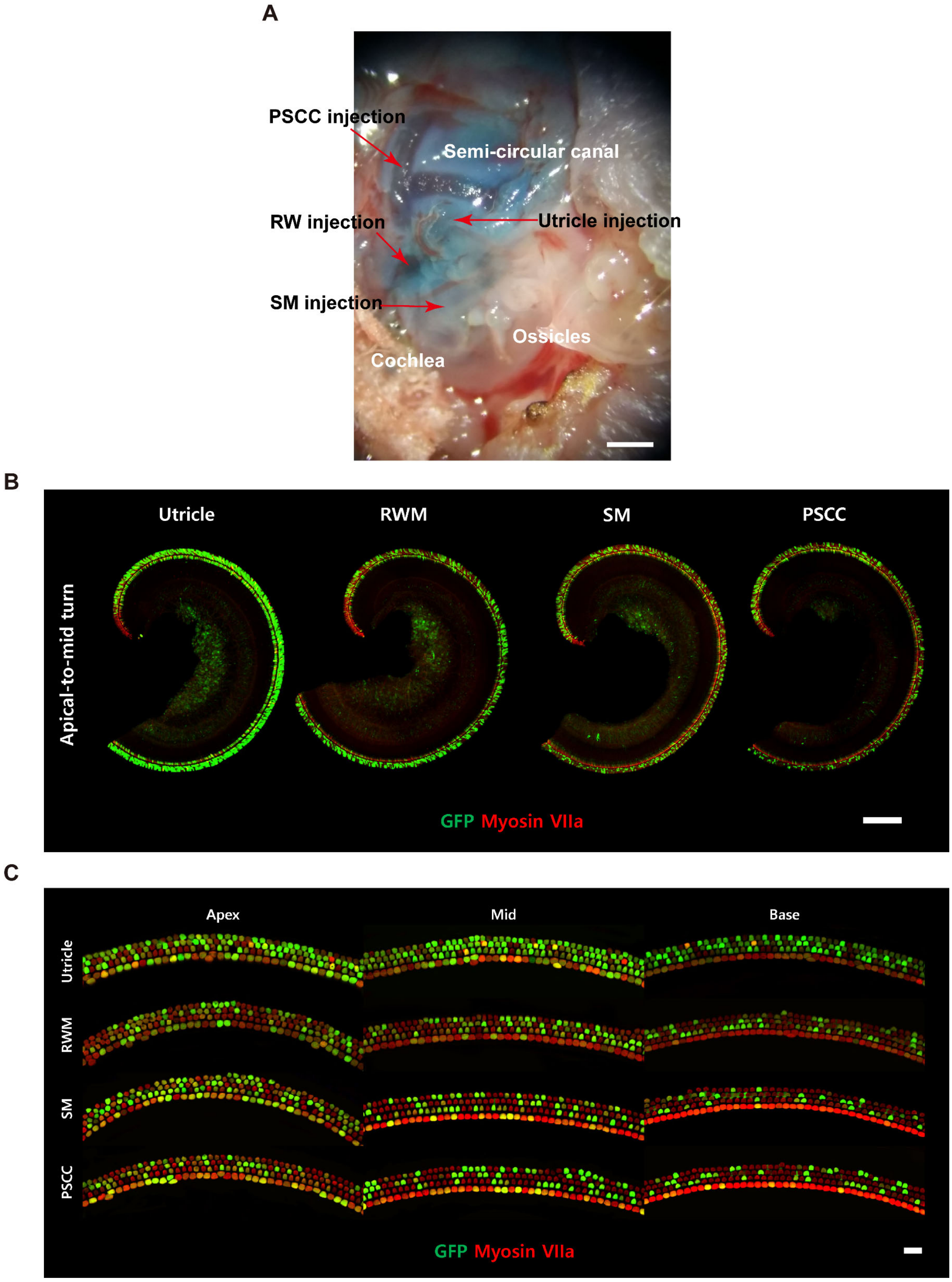

Figure S5. Optimization of delivery routes for *in vivo* gene editing.
(**A**) Post-auricular incision image of a pup at postnatal day 1 (P1) representing four injection routes, i.e., posterior semi-circular canal (PSCC), round window (RW), scala media (SM), and utricle. Methylene blue was injected as a marker. Scale bar, 500 µm. (**B**) Representative images of the apical-to-mid regions of a P7 wild-type pup injected with AAV2/Anc80L65-GFP at P1. Green, GFP; red, myo7a, a hair cell marker. Scale bar, 200 µm. (C) Representative images of apical, mid, basal regions of a P7 wild-type pup injected with AAV2/Anc80L65-GFP at P1. Green, GFP; red, myo7a, a hair cell marker. Scale bar, 20 µm.

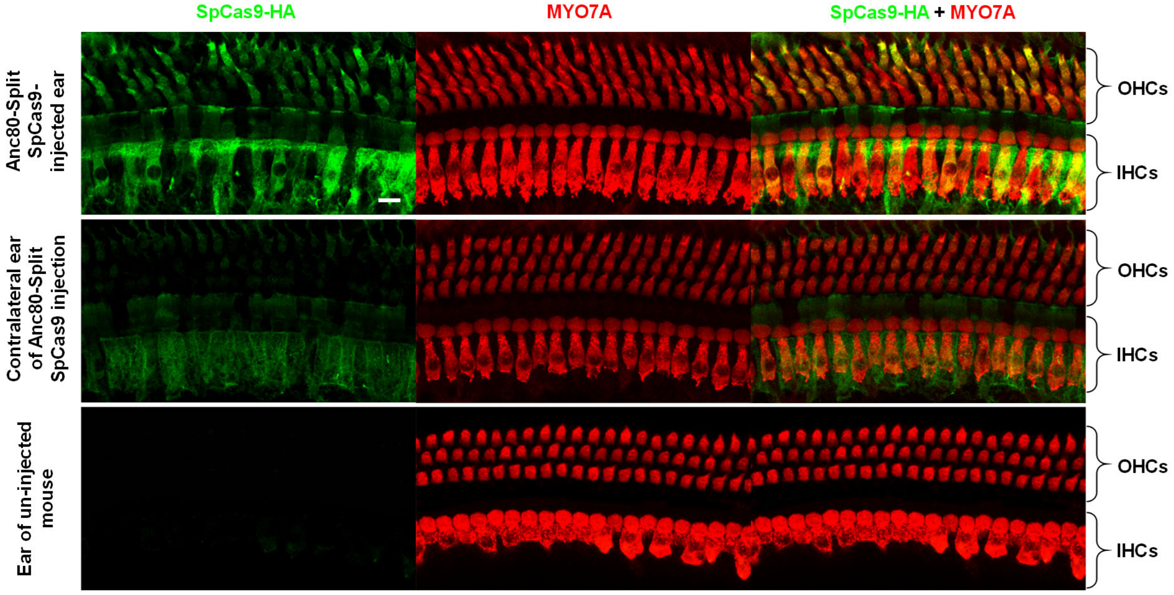

Figure S6. Transfection of SpCas9 in the cochlea hair cells.
Anc80-Split SpCas9 was injected via scala media of the cochlea at P2. Two weeks later, the injected (upper panel) and contralateral (mid panel) cochleae were harvested and immunostained with anti-HA antibody (SpCas9 with HA tag was used) to detect hair cell expression of SpCas9. For a negative control, ear of non-injected mouse was also stained with anti-HA antibody (lower panel). Green, SpCas9-HA; red, myo7a; OHCs, outer hair cells; IHCs, inner hair cells. Scale bar, 10 µm.

A

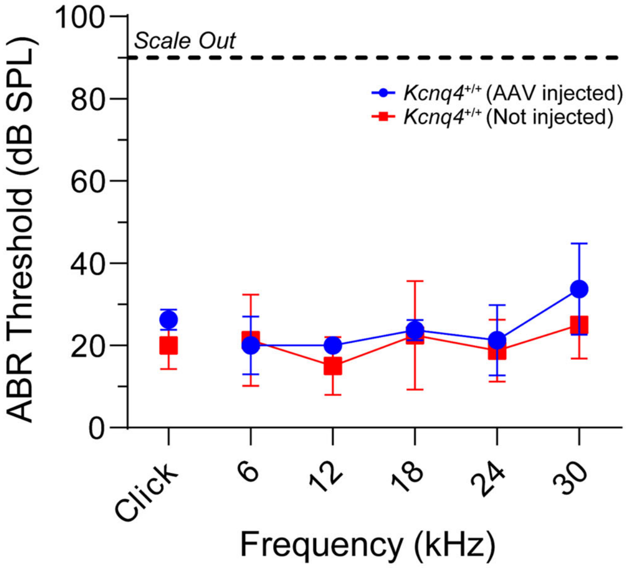

B

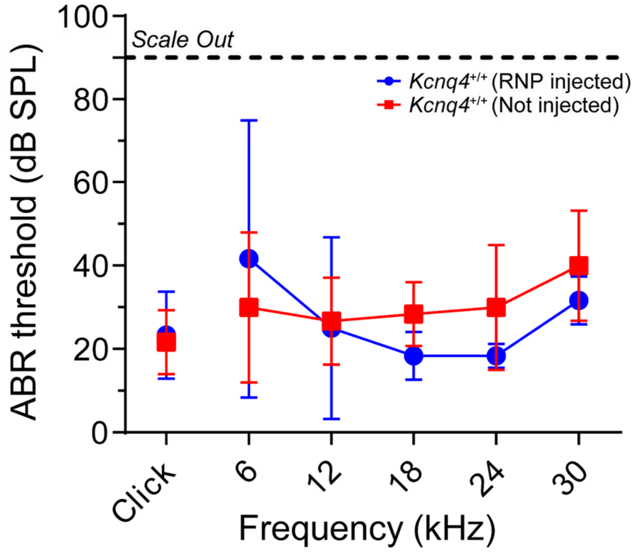

Figure S7. Safety of AAV and RNP injection into the scala media.
(A-B) Auditory brainstem response (ABR) threshold values measured in 3-week-old WT (*Kcnq4*$^{+/+}$) mice after AAV (A) and RNP (B) injection at P1. No significant differences were observed in the ABR threshold across all frequencies between RNP-injected (green) and contralateral non-injected (black) cochlea. Statistical analysis was performed using two-way ANOVA followed by Bonferroni's correction for multiple comparisons.

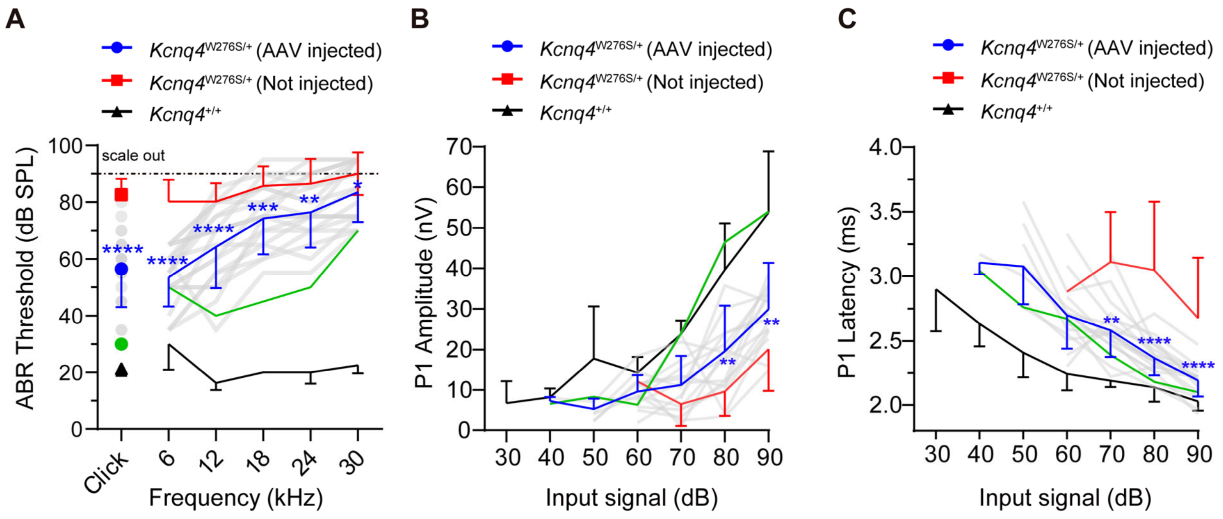

Figure S8. Characteristics of auditory brainstem responses (ABRs) in *Kcnq4*$^{W276S/+}$ after *in vivo* gene editing by AAV injection.

(**A**) ABR thresholds across different sound frequencies in *Kcnq4*$^{W276S/+}$ AAV-injected mice at age of 7 weeks are individually depicted in gray lines (n = 21); the best (green) and mean (blue) recovery traces are presented in AAV-injected mice. AAV-non-injected *Kcnq4*$^{W276S/+}$ (n = 21) and wild-type mice (n = 4) are depicted in red and black, respectively. (**B and C**) Peak 1 amplitudes (B) and latencies (C) measured from 6-kHz ABR waveforms as shown in (A). Statistical comparisons were performed between AAV-injected and non-injected mice. *$p < 0.05$, **$p < 0.01$, ***$p < 0.001$ and ****$p < 0.0001$ via two-way ANOVA followed by Bonferroni's correction for multiple comparisons.

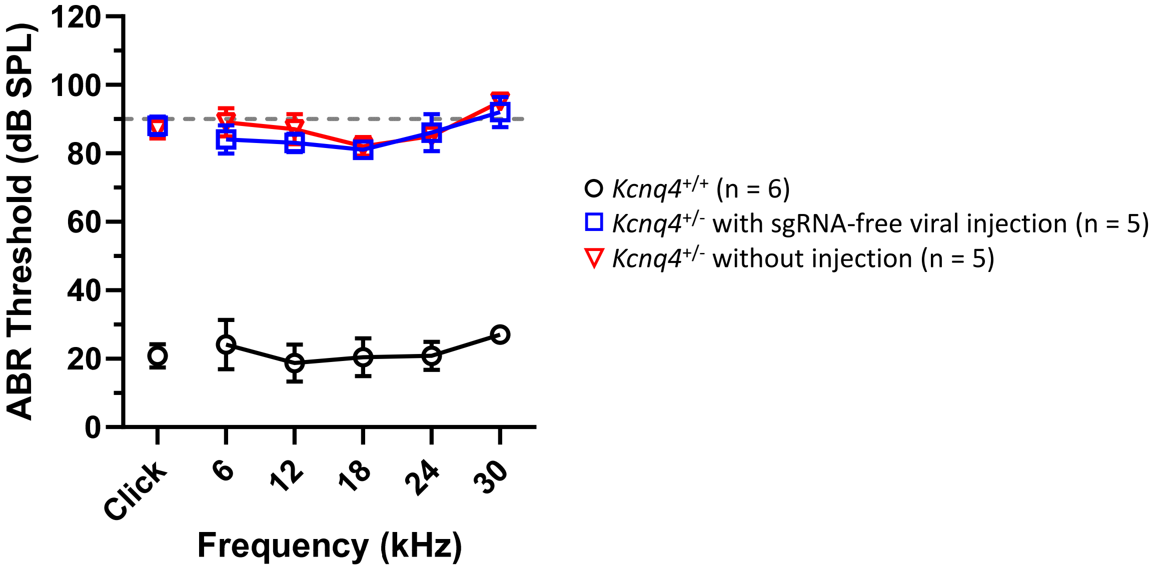

Figure S9. sgRNA-dependent hearing restoration by gene editing in *Kcnq4*$^{W276S/+}$ mouse.
To confirm whether hearing restoration by gene editing (shown in Figure 3) is dependent on targeted sgRNA (T3), we injected Anc80-Cas9 without sgRNA to *Kcnq4*$^{W276S/+}$ mice via scala media and measured hearing thresholds in ABR 7 weeks after injection. In comparison to the hearing of the non-injected ears of *Kcnq4*$^{W276S/+}$ mice, there was no difference in the hearing threshold between two groups, indicating that sgRNA is necessary to restore hearing function. $p > 0.05$ via two-way ANOVA followed by Bonferroni's correction for multiple comparisons.

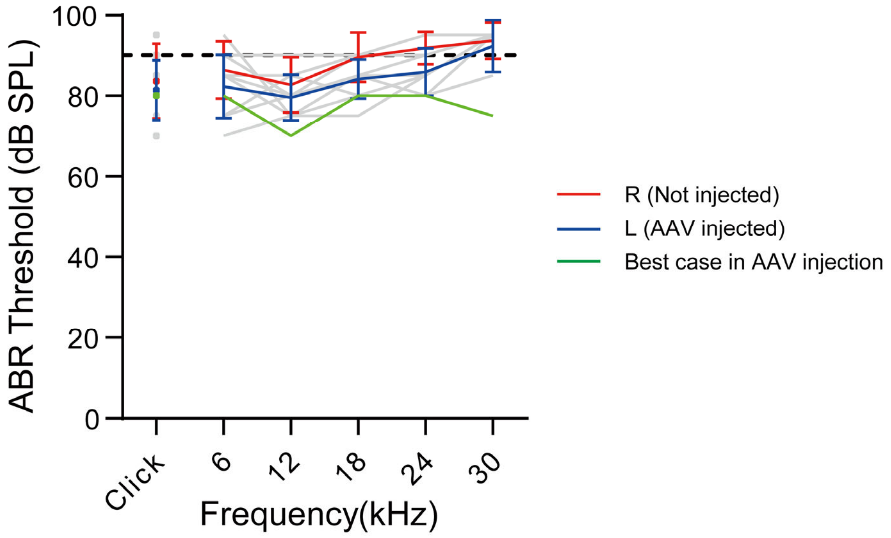

Figure S10. Long-term effect of *in vivo* gene editing in *Kcnq4*$^{W276S/+}$.
ABR thresholds at P77 (11 weeks) were compared between AAV-injected and non-injected ears. There was no significant difference between two groups. The best (green) and mean (blue) recovery traces are presented and compared to the threshold of non-injected ears (red). Statistical comparison was performed using two-way ANOVA with Bonferroni correction for multiple comparison.

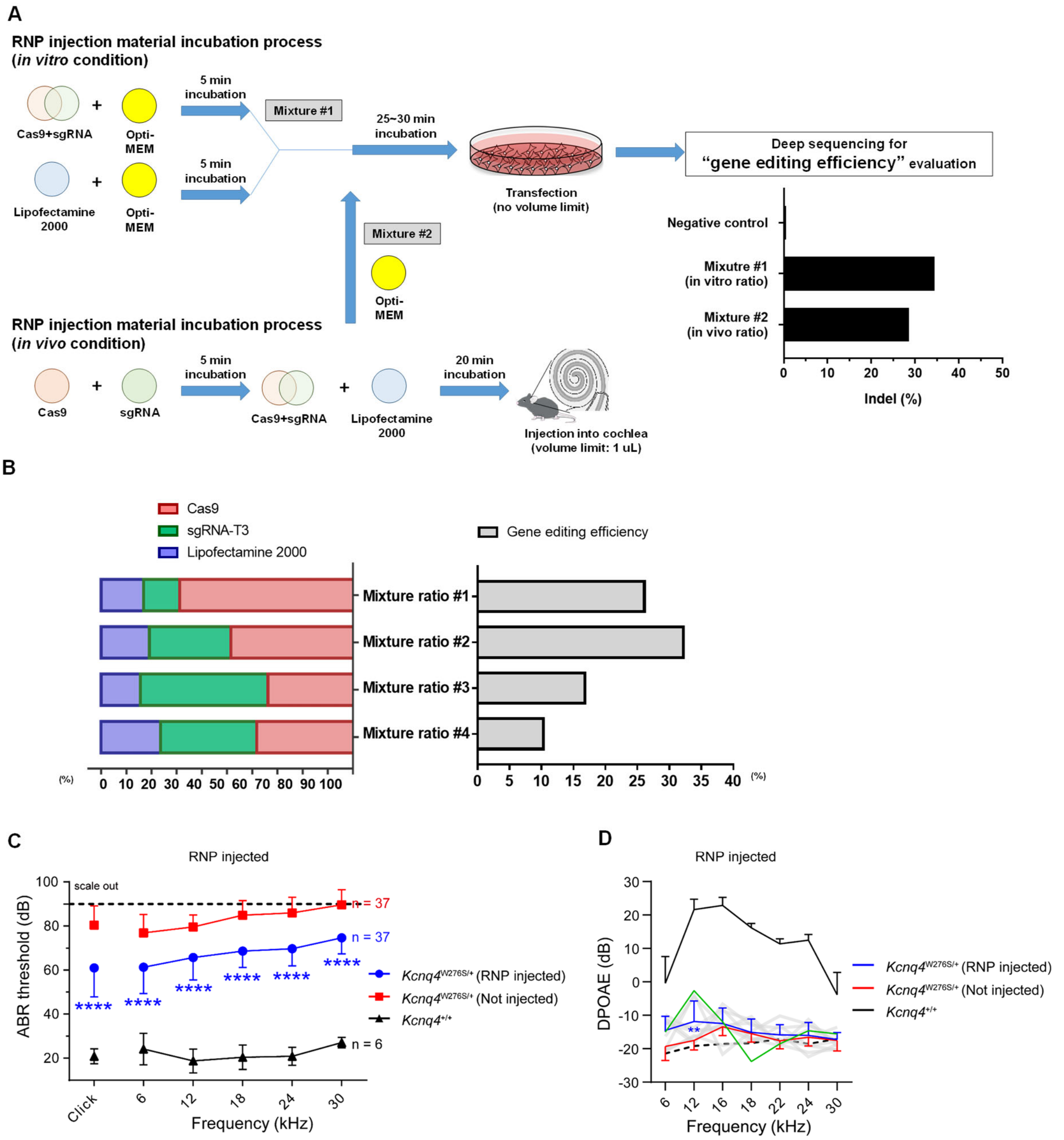

Figure S11. Optimization of ribonucleotide complex (RNP) vehicles and *in vivo* phenotypic rescue.
(**A**) Schematic diagram of the validation of the Cas9 and sgRNA complex with lipofectamine. *In vivo* injection of the RNP mixture was performed using two stepwise incubations. *In vitro* and *in vivo* RNP mixtures were transfected into HEK293 reporter cells with *Kcnq4* c.830G>C mutant alleles and validated for gene editing efficacy by deep sequencing, revealing 34.51% and 28.56% indel sequence fragments, respectively. For a negative control without any mixture, the indel sequence frequency was 0.5%. (**B**) *In vitro* optimization of the ratio of Cas9, sgRNA, and Lipofectamine 2000. RNP mixtures at various ratios were evaluated to identify the mixture with the highest efficacy by increasing the concentrations of Cas9 and sgRNA and the volume of Lipofectamine. (**C**) Comparative analysis of auditory brainstem response thresholds across all frequencies in RNP-injected mutant (n = 37), non-injected mutant (n = 37), and WT cochlea (n = 6). ****$p < 0.0001$ by two-way ANOVA followed by Bonferroni's correction for multiple comparisons. (**D**) Distortion-product otoacoustic emission thresholds of RNP-injected mutant (n = 10), non-injected mutant (n = 10), and WT mice (n = 4). The best (green) and mean (blue) corrected cochleae are presented with individual RNP-injected cochleae (gray). Dashed black line refers to the level of noise floor. **$p < 0.01$ via two-way ANOVA with Bonferroni's correction for multiple comparisons.

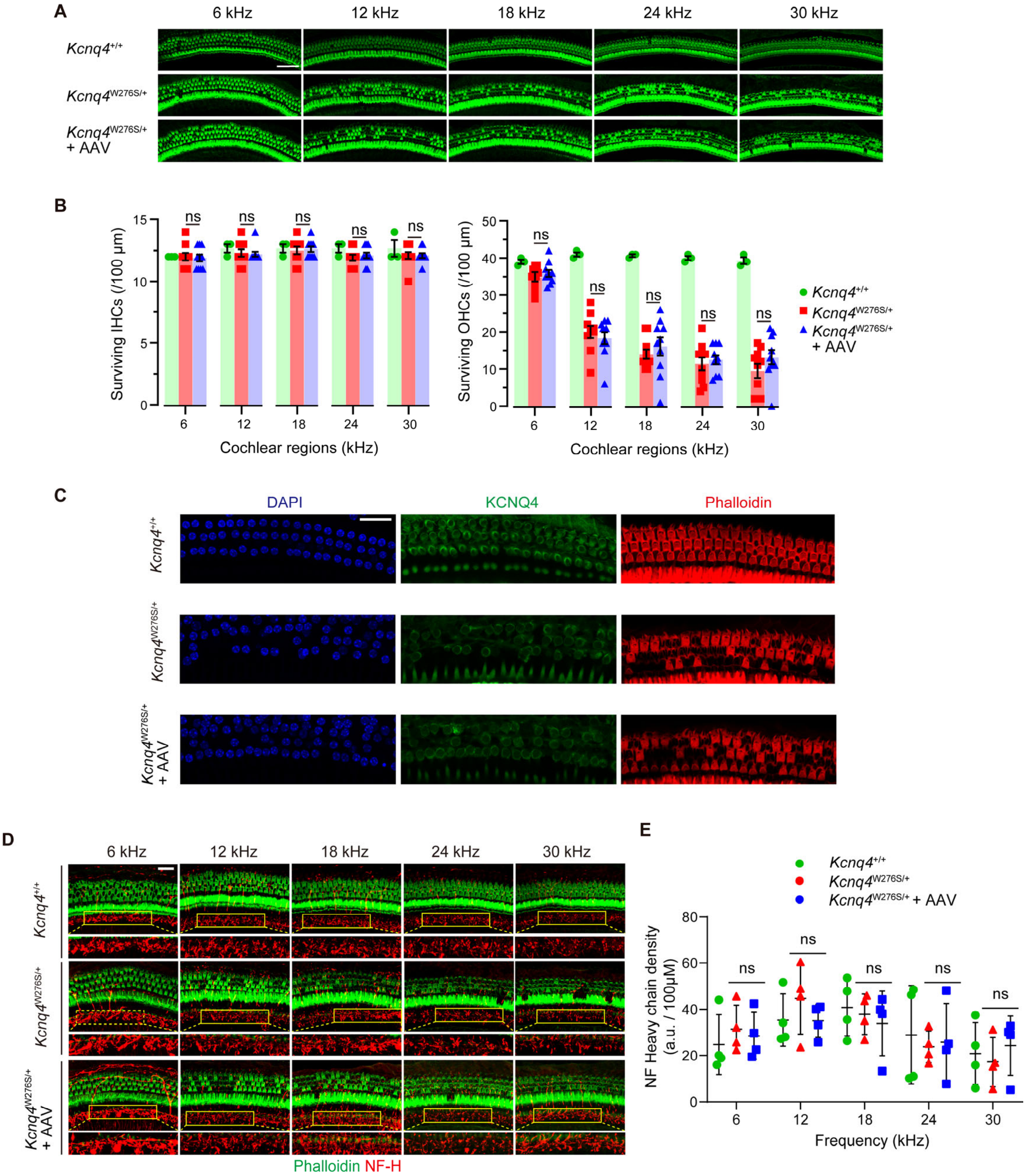

Figure S12. Effect of *in vivo* gene editing on the hair cells and neurofilaments of *Kcnq4*$^{W276S/+}$ mice.
(**A**) Hair cell survival patterns in cochlea of WT, non-injected mutant, and injected mutant 7-week-old mice across five frequencies after immunostaining with phalloidin (green). AAV, AAV injection; scale bar, 50 µm. (**B**) Numbers of live inner and outer hair cells in cochlea of WT, non-injected mutant, and injected mutant mice across five frequencies. ns, not significant by two-way ANOVA followed by Bonferroni's correction for multiple comparisons. (**C**) Whole-mount images of *Kcnq4*$^{+/+}$, non-injected *Kcnq4*$^{W276S/+}$, and AAV-injected *Kcnq4*$^{W276S/+}$ cochlea (in the 12-kHz region) from a 7-week-old mouse showing Kv7.4 (KCNQ4, green) expression in the outer hair cells. Scale bar, 20 µm. (**D**) Tonotopically mapped cochlear sections at 6-, 12-, 18-, 24-, and 30-kHz regions for WT (top), non-injected mutant (middle), and AAV-injected mutant (bottom) mice. Areas inside the yellow rectangle indicate 100-µm regions used to quantify the fluorescence density of the inner spiral plexus. Scale bar, 20 µm. (**E**) Quantitation of fluorescence density. No statistical significances were observed among the mouse groups (WT [black, n = 3], non-injected mutant [red, n = 4], and AAV-injected mutant [n = 4, blue]) in all frequency regions. ns, not significant via two-way ANOVA followed by Bonferroni's correction for multiple comparisons.

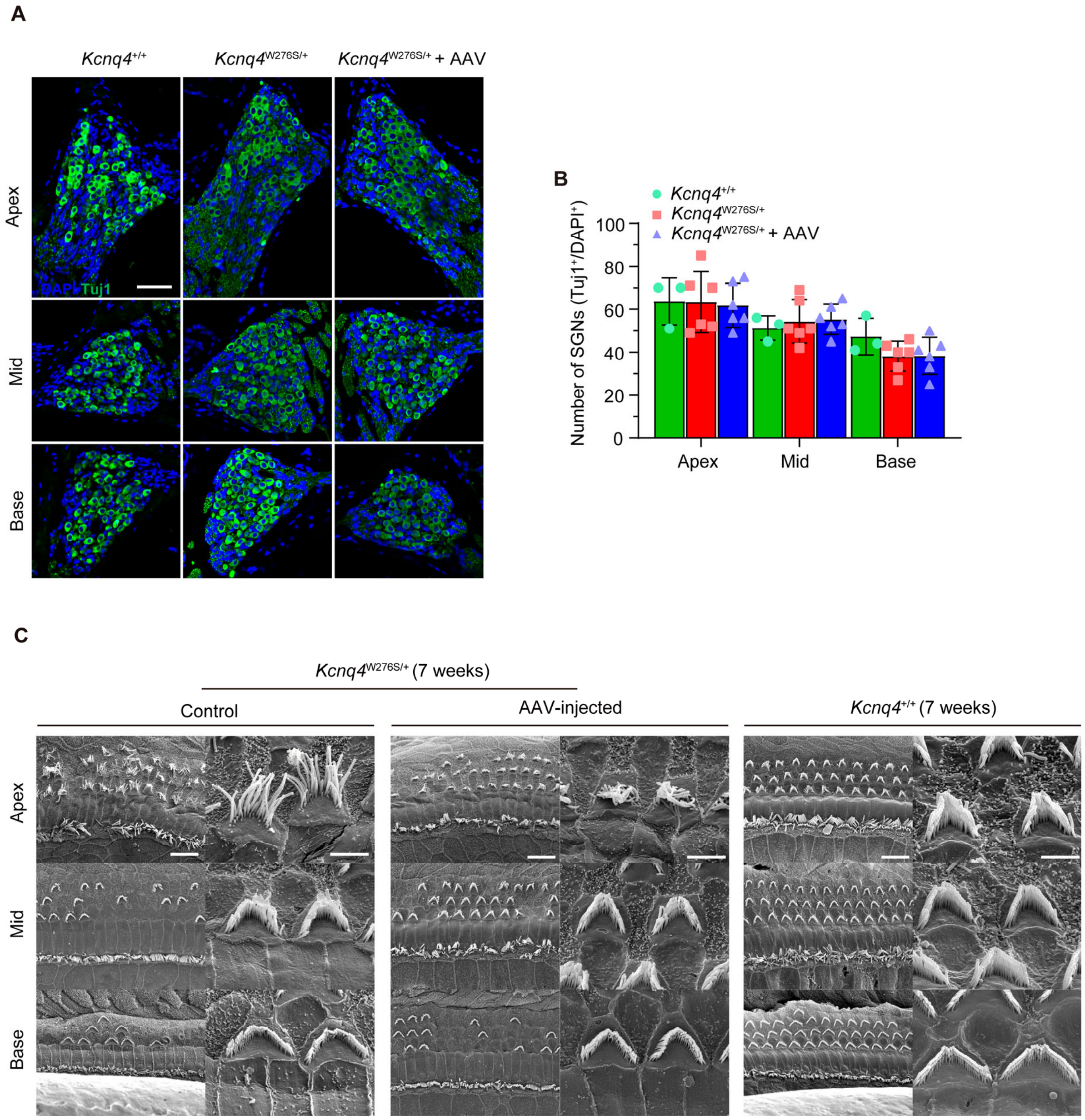

Figure S13. Effect of *in vivo* gene editing on neuronal survival and hair cell morphology in *Kcnq4*$^{\mathrm{W276S/+}}$ mice. (**A**) Cross-sectional images of the inner ear at the apex, mid, and base regions of cochlea of 7-week-old WT, non-injected mutant, and AAV-injected mutant mice showing ganglion survival. Tuj1 (green) positivity indicates alive spiral ganglion neurons (SGNs). Scale bar, 50 µm. (**B**) Quantitation of the number of alive SGNs according to tonotopic regions in (A). No significant differences were observed in the SGN survival rate among WT (green, $n = 3$), non-injected mutant (red, $n = 6$), and AAV-injected mutant (blue, $n = 6$) mice ($p > 0.05$ by two-way ANOVA followed by Bonferroni's correction for multiple comparisons). (**C**) Scanning electron micrographs at the apex, mid, and base from cochlea of 7-week-old non-injected mutant (control) and AAV-injected mutant mice. Scale bars, 20 µm (low power) and 5 µm (high power).

Supplemental Note 1. Coding sequences of dual AAV plasmid systems

Coding sequence of CMV-Cas9-Puro-2A-RFP

CMV enhancer colored in yellow, CMV promoter colored in pink, hSpCas9 is colored in blue, SV40 NLS colored in dark blue, HA colored in green E2A colored in dark green, TagRFP is colored in red, T2A is colored in dark grey, PuroR is colored in grey, and bGH poly A is colored in brown.

gacattgattattgactagttattaatagtaatcaattacggggtcattagttcatagcccatatatggagttccgcgttacataacttacggtaaatggcc
cgcctggctgaccgcccaacgacccccgcccattgacgtcaataatgacgtatgttcccatagtaacgccaatagggactttccattgacgtcaatg
ggtggactatttacggtaaactgcccacttggcagtacatcaagtgtatcatatgccaagtacgccccctattgacgtcaatgacggtaaatggcccg
cctggcattatgcccagtacatgaccttatgggactttcctacttggcagtacatctacgtattagtcatcgctattaccatggtgatgcggttttggcagta
catcaatgggcgtggatagcggtttgactcacggggatttccaagtctccaccccattgacgtcaatgggagtttgttttggcaccaaaatcaacggg
actttccaaaatgtcgtaacaactccgccccattgacgcaaatgggcggtaggcgtgtacggtgggaggtctatataagcagagctctctggctaact
agagaacccactgcttactggcttatcgaaattaatacgactcactatagggagacccaagcttgccaccatggacaagaagtacagcatcggcct
ggacatcggtaccaacagcgtgggctgggccgtgatcaccgacgagtacaaggtgcccagcaagaagttcaaggtgctgggcaacaccgacc
gccacagcatcaagaagaacctgatcggcgccctgctgttcgacagcggcgagaccgccgaggccacccgcctgaagcgcaccgcccgccg
ccgctacacccgccgcaagaaccgcatctgctacctgcaggagatcttcagcaacgagatggccaaggtggacgacagcttcttccaccgcctgg
aggagagcttcctggtggaggaggacaagaagcacgagcgccaccccatcttcggcaacatcgtggacgaggtggcctaccacgagaagtac
cccaccatctaccacctgcgcaagaagctggtggacagcaccgacaaggccgacctgcgcctgatctacctggccctggcccacatgatcaagtt
ccgcggccacttcctgatcgagggcgacctgaaccccgacaacagcgacgtggacaagctgttcatccagctggtgcagacctacaaccagctg
ttcgaggagaaccccatcaacgccagcggcgtggacgccaaggccatcctgagcgcccgcctgagcaagagccgccgcctggagaacctgat
cgcccagctgcccggcgagaagaagaacggcctgttcggcaacctgatcgccctgagcctgggcctgacccccaacttcaagagcaacttcgac
ctggccgaggacgccaagctgcagctgagcaaggacacctacgacgacgacctggacaacctgctggcccagatcggcgaccagtacgccg
acctgttcctggccgccaagaacctgagcgacgccatcctgctgagcgacatcctgcgcgtgaacaccgagatcaccaaggcccccctgagcgc
cagcatgatcaagcgctacgacgagcaccaccaggacctgaccctgctgaaggccctggtgcgccagcagctgcccgagaagtacaaggaga
tcttcttcgaccagagcaagaacggctacgccggctacatcgacggcggcgccagccaggaggagttctacaagttcatcaagcccatcctggag
aagatggacggcaccgaggagctgctggtgaagctgaaccgcgaggacctgctgcgcaagcagcgcaccttcgacaacggcagcatcccca
ccagatccacctgggcgagctgcacgccatcctgcgccgccaggaggacttctaccccttcctgaaggacaaccgcgagaagatcgagaagat
cctgaccttccgcatcccctactacgtgggccccctggcccgcggcaacagccgcttcgcctggatgacccgcaagagcgaggagaccatcacc
ccctggaacttcgaggaggtggtggacaagggcgccagcgcccagagcttcatcgagcgcatgaccaacttcgacaagaacctgcccaacgag
aaggtgctgcccaagcacagcctgctgtacgagtacttcaccgtgtacaacgagctgaccaaggtgaagtacgtgaccgagggcatgcgcaagc
ccgccttcctgagcggcgagcagaagaaggccatcgtggacctgctgttcaagaccaaccgcaaggtgaccgtgaagcagctgaaggaggact
acttcaagaagatcgagtgcttcgacagcgtggagatcagcggcgtggaggaccgcttcaacgccagcctgggcacctaccacgacctgctgaa
gatcatcaaggacaaggacttcctggacaacgaggagaacgaggacatcctggaggacatcgtgctgaccctgaccctgttcgaggaccgcga
gatgatcgaggagcgcctgaagacctacgcccacctgttcgacgacaaggtgatgaagcagctgaagcgccgccgctacaccggctggggccg
cctgagccgcaagcttatcaacggcatccgcgacaagcagagcggcaagaccatcctggacttcctgaagagcgacggcttcgccaaccgcaa
cttcatgcagctgatccacgacgacagcctgaccttcaaggaggacatccagaaggcccaggtgagcggccagggcgacagcctgcacgagc
acatcgccaacctggccggcagccccgccatcaagaagggcatcctgcagaccgtgaaggtggtggacgagctggtgaaggtgatgggccgc
cacaagcccgagaacatcgtgatcgagatggcccgcgagaaccagaccacccagaagggccagaagaacagccgcgagcgcatgaagcg
catcgaggagggcatcaaggagctgggcagccagatcctgaaggagcaccccgtggagaacacccagctgcagaacgagaagctgtacctgt
actacctgcagaacggccgcgacatgtacgtggaccaggagctggacatcaaccgcctgagcgactacgacgtggaccacatcgtgccccaga
gcttcctgaaggacgacagcatcgacaacaaggtgctgacccgcagcgacaagaaccgcggcaagagcgacaacgtgcccagcgaggagg
tggtgaagaagatgaagaactactggcgccagctgctgaacgccaagctgatcacccagcgcaagttcgacaacctgaccaaggccgagcgc
ggcggcctgagcgagctggacaaggccggcttcatcaagcgccagctggtggagacccgccagatcaccaagcacgtggcccagatcctgga
cagccgcatgaacaccaagtacgacgagaacgacaagctgatccgcgaggtgaaggtgatcaccctgaagagcaagctggtgagcgacttcc
gcaaggacttccagttctacaaggtgcgcgagatcaacaactaccaccacgcccacgacgcctacctgaacgccgtggtgggcaccgccctgat
caagaagtaccccaagctggagagcgagttcgtgtacggcgactacaaggtgtacgacgtgcgcaagatgatcgccaagagcgagcaggaga
tcggcaaggccaccgccaagtacttcttctacagcaacatcatgaacttcttcaagaccgagatcaccctggccaacggcgagatccgcaagcgc
cccctgatcgagaccaacggcgagaccggcgagatcgtgtgggacaagggccgcgacttcgccaccgtgcgcaaggtgctgagcatgcccca
ggtgaacatcgtgaagaagaccgaggtgcagaccggcggcttcagcaaggagagcatcctgcccaagcgcaacagcgacaagctgatcgcc
cgcaagaaggactgggaccccaagaagtacggcggcttcgacagccccaccgtggcctacagcgtgctggtggtggccaaggtggagaaggg
caagagcaagaagctgaagagcgtgaaggagctgctgggcatcaccatcatggagcgcagcagcttcgagaagaaccccatcgacttcctgga
ggccaagggctacaaggaggtgaagaaggacctgatcatcaagctgcccaagtacagcctgttcgagctggagaacggccgcaagcgcatgct

ggccagcgccggcgagctgcagaagggcaacgagctggccctgcccagcaagtacgtgaacttcctgtacctggccagccactacgagaagct
gaagggcagccccgaggacaacgagcagaagcagctgttcgtggagcagcacaagcactacctggacgagatcatcgagcagatcagcgag
ttcagcaagcgcgtgatcctggccgacgccaacctggacaaggtgctgagcgcctacaacaagcaccgcgacaagcccatccgcgagcaggc
cgagaacatcatccacctgttcaccctgaccaacctgggcgcccccgccgccttcaagtacttcgacaccaccatcgaccgcaagcgctacacca
gcaccaaggaggtgctggacgccaccctgatccaccagagcatcaccggtctgtacgagacccgcatcgacctgagccagctgggcggcgac
ggcggctccggacctccaaagaaaaagagaaaagtatacccctacgacgtgcccgactacgccgaattcggaagcgggcagtgcaccaacta
cgccctgctgaagctggccggcgacgtggagagcaaccccggccccggatccatggtgtctaagggcgaagagctgattaaggagaacatgca
catgaagctgtacatggagggcaccgtgaacaaccaccacttcaagtgcacatccgagggcgaaggcaagccctacgagggcacccagacca
tgagaatcaaggtggtcgagggcggccctctcccttcgccttcgacatcctggctaccagcttcatgtacggcagcagaaccttcatcaaccacac
ccagggcatccccgacttctttaagcagtccttccctgagggcttcacatgggagagagtcaccacatacgaggacgggggcgtgctgaccgctac
ccaggacaccagcctccaggacggctgcctcatctacaacgtcaagatcagaggggtgaacttcccatccaacggccctgtgatgcagaagaaa
acactcggctgggaggccaacaccgagatgctgtaccccgctgacggcggcctggaaggcagaagcgacatggccctgaagctcgtgggcgg
gggccacctgatctgcaacttcaagaccacatacagatccaagaaacccgctaagaacctcaagatgcccggcgtctactatgtggaccacaga
ctggaaagaatcaaggaggccgacaaagagacctacgtcgagcagcacgaggtggctgtggccagatactgcgacctccctagcaaactggg
gcacaaacttaatggctccgagggcagaggaagccttctaacatgcggtgacgtggaggagaatcccggcccttccgggatgaccgagtacaag
cccacggtgcgcctcgccacccgcgacgacgtccccagggccgtacgcaccctcgccgccgcgttcgccgactaccccgccacgcgccacacc
gtcgatccagaccgccacatcgagcgggtcaccgagctgcaagaactcttcctcacgcgcgtcgggctcgacatcggcaaggtgtgggtcgcgg
acgacggcgccgcggtggcggtctggaccacgccggagagcgtcgaagcgggggcggtgttcgccgagatcggcccgcgcatggccgagttg
agcggttcccggctggccgcgcagcaacagatggaaggtctcctggcgccgcaccggcccaaggagcccgcgtggttcctggccaccgtcggc
gtctcgcccgaccaccagggcaagggtctgggcagcgccgtcgtgctccccggagtggaggcggccgagcgcgccggggtgcccgccttcctg
gagacctccgcgccccgcaacctccccttctacgagcggctcggcttcaccgtcaccgccgacgtcgaggtgcccgaaggaccgcgcacctggt
gcatgacccgcaagcccggtgcctgacatgcatctagagggccctattctatagtgtcacctaaatgctagagctcgctgatcagcctcgactgtgcc
ttctagttgccagccatctgttgtttgcccctcccccgtgccttccttgaccctggaaggtgccactcccactgtcctttcctaataaaatgaggaaattgca
tcgcattgtctgagtaggtgtcattctattctggggggtggggtggggcaggacagcaagggggaggattgggaagacaatagcaggcatgctgg
ggatgcggtgggctctatgg

Coding sequence of U6-pRG2-sgRNA

U6 colored in yellow, BsaI restriction enzyme cloning site is colored in pink, gRNA scaffold colored in dark green and pUC ori is colored in green

gagggcctatttcccatgattccttcatatttgcatatacgatacaaggctgttagagagataattggaattaatttgactgtaaacacaaagatattagta
caaaatacgtgacgtagaaagtaataatttcttgggtagtttgcagttttaaaattatgttttaaaatggactatcatatgcttaccgtaacttgaaagtattt
cgatttcttggctttatatatcttgtggaaaggacgaaacaccggtgagaccggatccggtctccgttttagagctagaaatagcaagttaaaataagg
ctagtccgttatcaacttgaaaaagtggcaccgagtcggtgcttttttctagattcgcgatgtacgggccagatatacgcgttgacattgattattgactag
ttgtcttcctgcattaatgaatcggccaacgcgcggggagaggcggtttgcgtattgggcgctcttccgcttcctcgctcactgactcgctgcgctcggtc
gttcggctgcggcgagcggtatcagctcactcaaaggcggtaatacggttatccacagaatcaggggataacgcaggaaagaacatgtgagcaa
aaggccagcaaaaggccaggaaccgtaaaaaggccgcgttgctggcgtttttccataggctccgcccccctgacgagcatcacaaaaatcgacg
ctcaagtcagaggtggcgaaacccgacaggactataaagataccaggcgtttccccctggaagctccctcgtgcgctctcctgttccgaccctgccg
cttaccggatacctgtccgcctttctcccttcgggaagcgtggcgctttctcaatgctcacgctgtaggtatctcagttcggtgtaggtcgttcgctccaag
ctgggctgtgtgcacgaaccccccgttcagcccgaccgctgcgccttatccggtaactatcgtcttgagtccaacccggtaagacacgacttatcgcc
actggcagcagccactggtaacaggattagcagagcgaggtatgtaggcggtgctacagagttcttgaagtggtggcctaactacggctacactag
aaggacagtatttggtatctgcgctctgctgaagccagttaccttcggaaaaagagttggtagctcttgatccggcaaacaaaccaccgctggtagc
ggtggtttttttgtttgcaagcagcagattacgcgcagaaaaaaaggatctcaa

Coding sequence of U6-sgRNA-split–N-Cas9 N-intein

AAV2 ITR is colored in red, U6 colored in dark green, Kcnq4-sgRNA T3 is colored in violet, gRNA scaffold is colored in grey, CMV enhancer colored in yellow, CMV promoter colored in pink, SV40 NLS colored in dark blue, FLAG-tag colored in green, split–N-Cas9 is colored in blue, N-Intein is colored in dark grey, and bGH poly A is colored in brown.

cctgcaggcagctgcgcgctcgctcgctcactgaggccgcccgggcaaagcccgggcgtcgggcgacctttggtcgcccggcctcagtgagcga
gcgagcgcgcagagagggagtggccaactccatcactaggggttcctgcggccgcacgcgtgagggcctatttcccatgattccttcatatttgcat
atacgatacaaggctgttagagagataattggaattaatttgactgtaaacacaaagatattagtacaaaatacgtgacgtagaaagtaataatttctt
gggtagtttgcagttttaaaattatgttttaaaatggactatcatatgcttaccgtaacttgaaagtatttcgatttcttggctttatatatcttGTGGAAAG

GACGAAACACCGCGACTCGCTCTGGTCGGGGAgttttagagctaGAAAtagcaagttaaaataaggctagtccgttatcaa
cttgaaaaagtggcaccgagtcggtgcTTTTTTtctagactcgaggcgttgacattgattattgactagttattaatagtaatcaattacggggtcatt
agttcatagcccatatatggagttccgcgttacataacttacggtaaatggcccgcctggctgaccgcccaacgacccccgcccattgacgtcaata
atgacgtatgttcccatagtaacgccaatagggactttccattgacgtcaatgggtggagtatttacggtaaactgcccacttggcagtacatcaagtgt
atcatatgccaagtacgccccctattgacgtcaatgacggtaaatggcccgcctggcattatgcccagtacatgaccttatgggactttcctacttggca
gtacatctacgtattagtcatcgctattaccatggtgatgcggttttggcagtacatcaatgggcgtggatagcggtttgactcacggggatttccaagtct
ccaccccattgacgtcaatgggagtttgttttggcaccaaaatcaacgggactttccaaaatgtcgtaacaactccgccccattgacgcaaatgggc
ggtaggcgtgtacggtgggaggtctatataagcagagctctctggctaactaccggtgccaccatgggacctaagaaaaagaggaaggtggcgg
ccgctgactacaaggatgacgacgataaaggtatccacggagtcccagcagccgacaagaagtacagcatcggcctggacatcggcaccaac
tctgtgggctgggccgtgatcaccgacgagtacaaggtgcccagcaagaaattcaaggtgctgggcaacaccgaccggcacagcatcaagaag
aacctgatcggagccctgctgttcgacagcggcgaaacagccgaggccacccggctgaagagaaccgccagaagaagatacaccagacgg
aagaaccggatctgctatctgcaagagatcttcagcaacgagatggccaaggtggacgacagcttcttccacagactggaagagtccttcctggtg
gaagaggataagaagcacgagcggcaccccatcttcggcaacatcgtggacgaggtggcctaccacgagaagtaccccaccatctaccacctg
agaaagaaactggtggacagcaccgacaaggccgacctgcggctgatctatctggccctggcccacatgatcaagttccggggccacttcctgat
cgagggcgacctgaaccccgacaacagcgacgtggacaagctgttcatccagctggtgcagacctacaaccagctgttcgaggaaaaccccat
caacgccagcggcgtggacgccaaggccatcctgtctgccagactgagcaagagcagacggctggaaaatctgatcgcccagctgcccggcg
agaagaagaatggcctgttcggcaacctgattgccctgagcctgggcctgacccccaacttcaagagcaacttcgacctggccgaggatgccaaa
ctgcagctgagcaaggacacctacgacgacgacctggacaacctgctggcccagatcggcgaccagtacgccgacctgtttctggccgccaag
aacctgtccgacgccatcctgctgagcgacatcctgagagtgaacaccgagatcaccaaggcccccctgagcgcctctatgatcaagagatacg
acgagcaccaccaggacctgaccctgctgaaagctctcgtgcggcagcagctgcctgagaagtacaaagagattttcttcgaccagagcaagaa
cggctacgccggctacattgacggcggagccagccaggaagagttctacaagttcatcaagcccatcctggaaaagatggacggcaccgagga
actgctcgtgaagctgaacagagaggacctgctgcggaagcagcggaccttcgacaacggcagcatcccccaccagatccacctgggagagct
gcacgccattctgcggcggcaggaagatttttacccattcctgaaggacaaccgggaaaagatcgagaagatcctgaccttccgcatcccctacta
cgtgggccctctggccaggggaaacagcagattcgcctggatgaccagaaagagcgaggaaaccatcaccccctggaacttcgaggaagtggt
ggacaagggcgcttccgcccagagcttcatcgagcggatgaccaacttcgataagaacctgcccaacgagaaggtgctgcccaagcacagcct
gctgtacgagtacttcaccgtgtataacgagctgaccaaagtgaaatacgtgaccgagggaatgagaaagcccgccttcctgagcggcgagcag
aaaaaggccatcgtggacctgctgttcaagaccaaccggaaagtgaccgtgaagcagctgaaagaggactacttcaagaaaatcgagtgcctgt
cctacgagaccgaaatcctgaccgtggagtatgggctgctgcccatcggcaagattgtggagaagcggattgaatgcaccgtgtatagcgtggac
aacaacggcaacatctacacccagcccgtggctcagtggcacgacaggggcgagcaggaggtgtttgagtattgtctggaggacggcagcctga
ttagagccaccaaagaccacaagttcatgaccgtggacgggcagatgctgcccattgacgagatttttgagcgggaacttgacctgatgcgggtgg
acaacctgcccaacgaattcgatatcaagcttatcgataccgagcgctgctcgagagatctacgggtggcatccctgtgacccctccccagtgcctct
cctggccctggaagttgccactccagtgcccaccagccttgtcctaataaaattaagttgcatcattttgtctgactaggtgtccttctataatattatgggg
tggaggggggtggtatggagcaaggggcaagttgggaagacaacctgtagggcctgcggggtctattgggaaccaagctggagtgcagtggca
caatcttggctcactgcaatctccgcctcctgggttcaagcgattctcctgcctcagcctcccgagttgttgggattccaggcatgcatgaccaggctca
gctaatttttgtttttttggtagagacggggtttcaccatattggccaggctggtctccaactcctaatctcaggtgatctacccaccttggcctcccaaattg
ctgggattacaggcgtgaaccactgctcccttccctgtccttctgattttgtaggtaaccacgtgcggaccgagcggccgcaggaacccctagtgatg
gagttggccactccctctctgcgcgctcgctcgctcactgaggccgggcgaccaaaggtcgcccgacgcccgggctttgcccgggcggcctcagt
gagcgagcgagcgcgcagctgcctgcagg

Caption for Supplemental Movie 1

***Ex vivo* imaging of outer hair cells using thallium-sensitive dye (FluxOR-Tl^{+}) in the apical turn (6 kHz) of a wild-type cochlea.** The videos were captured from 0 to 120 s after the reference time of the addition of thallium in the buffer and reframed in 30 s. Colors refer to FluxOR-Tl^{+} intensity, which is correlated with the membrane potential set by electro-chemical gradients with potassium, sodium, and chloride. Purple indicates low intensity, red indicates high intensity.
